# Site Diversity and Mechanism of Metal‐Exchanged Zeolite Catalyzed Non‐Oxidative Propane Dehydrogenation

**DOI:** 10.1002/advs.202207756

**Published:** 2023-03-10

**Authors:** Yong Yuan, Zhaoqi Zhao, Raul F. Lobo, Bingjun Xu

**Affiliations:** ^1^ Center for Catalytic Science and Technology Department of Chemical and Biomolecular Engineering University of Delaware Newark DE 19716 USA; ^2^ College of Chemistry and Molecular Engineering Peking University Beijing 100871 China

**Keywords:** active structure, Ga/H‐ZSM‐5, metal‐exchanged zeolite catalysts, propane dehydrogenation, reaction mechanism

## Abstract

Metal‐exchanged zeolites are well‐known propane dehydrogenation (PDH) catalysts; however, the structure of the active species remains unresolved. In this review, existing PDH catalysts are first surveyed, and then the current understanding of metal‐exchanged zeolite catalysts is described in detail. The case of Ga/H‐ZSM‐5 is employed to showcase that advances in the understanding of structure–activity relations are often accompanied by technological or conceptional breakthroughs. The understanding of Ga speciation at PDH conditions has evolved owing to the advent of in situ/operando characterizations and to the realization that the local coordination environment of Ga species afforded by the zeolite support has a decisive impact on the active site structure. In situ/operando quantitative characterization of catalysts, rigorous determination of intrinsic reaction rates, and predictive computational modeling are all significant in identifying the most active structure in these complex systems. The reaction mechanism could be both intricately related to and nearly independent of the details of the assumed active structure, as in the two main proposed PDH mechanisms on Ga/H‐ZSM‐5, that is, the carbenium mechanism and the alkyl mechanism. Perspectives on potential approaches to further elucidate the active structure of metal‐exchanged zeolite catalysts and reaction mechanisms are discussed in the final section.

## Graphical abstract

Metal‐exchanged zeolites are well‐known alkane dehydrogenation catalysts. In this review, key technological or conceptional breakthroughs in the understanding of structure–activity relations in propane dehydrogenation on metal‐exchanged zeolites are summarized by employing Ga/H‐ZSM‐5 as an example. In situ/operando quantitative characterization, rigorous determination of intrinsic rates, and predictive computational modeling are all instrumental in identifying the most active structure.

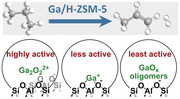

## 1 Introduction

### 1.1 Background

Propylene is one of the main feedstocks in the chemical industry, used in the production of a great number of chemicals such as polypropylene, acrylonitrile, propylene oxide, and cymene.^[^ ^1^ ^]^ Propylene is primarily a by‐product of steam cracking (SC) and the fluid catalytic cracking (FCC) of naphtha.^[^ ^2^ ^]^ In 2015, SC and FCC combined accounted for 81% of global propylene production capacity.^[^ ^3^, ^4^ ^]^ The annual propylene production capacity in 2019 was about 130 megatons, and the demand is expected to reach 191 megatons by 2030,^[^ ^5^ ^]^ laying bare a growing gap between the supply and demand of propylene.^[^ ^6^ ^]^ In addition to SC and FCC, several on‐purpose propylene technologies have been developed to increase propylene production including propane dehydrogenation (PDH), olefin metathesis, and methanol‐to‐propylene. The rapid increase in shale gas production—with its substantial content of natural gas liquids^[^ ^6^ ^]^ and thus propane^[^ ^5^ ^]^—has led to increased interest in PDH: it currently accounts for ≈10% of global propylene production.^[^ ^7^ ^]^

PDH can be broadly grouped into two categories: non‐oxidative and oxidative. In non‐oxidative PDH, propane is catalytically dehydrogenated to produce stoichiometric amounts of propylene and hydrogen. It is endothermic ($\Delta H_{298\;K}^{\theta }$ = 124.3 kJ mol^−1^) and a volume‐increasing reaction (C_3_H_8_ → C_3_H_6_ + H_2_), and thus is favored at high temperature and low pressure.^[^ ^8^, ^9^ ^]^ To achieve significant propylene yields, PDH typically operates at 550–700 °C. Common side reactions include C—C bond cleavage via cracking and hydrogenolysis: C_3_H_8_ → CH_4_ + C_2_H_4_ ($\Delta H_{298\;K}^{\theta }$ = 98.9 kJ mol^−1^), C_3_H_8_ + H_2_ → CH_4_ + C_2_H_6_ ($\Delta H_{298\;K}^{\theta }$ = −37.7 kJ mol^−1^), and deep dehydrogenation leading to the coke formation: C_3_H_8_ → 3C + 4H_2_ ($\Delta H_{298\;K}^{\theta }$ = 119.5 kJ mol^−1^).^[^ ^9^ ^]^ Non‐oxidative PDH typically exhibits good selectivity for propylene (generally >90%), but low propylene yields (typically < 50%) due to the unfavorable thermodynamics and catalyst deactivation are an obstacle to further market penetration.

Oxidants, usually O_2_, can be co‐fed with propane to enhance the thermodynamic driving force in the oxidative dehydrogenation of propane (ODHP) process. This process is exothermic ($\Delta H_{298\;K}^{\theta }$ = −117 kJ mol^−1^) and thus the conversion of propane is no longer limited by thermodynamics. In addition, coke formation is inhibited by the presence of a strong oxidant, leading to better catalyst stability. Deep oxidation of propane to CO and CO_2_ is the main challenge in catalyst design for the ODHP process.^[^ ^5^ ^]^ Vanadium‐based materials have been the most extensively investigated catalysts in ODHP,^[^ ^5^ ^]^ but B, Co, Cr, Fe, and Ni‐based materials have also been explored.^[^ ^5^, ^10^, ^11^, ^12^ ^]^ For example, hexagonal boron nitride achieves 79% propylene selectivity at 490 °C with C_3_H_8_:O_2_ = 2:1 (14% propane conversion).^[^ ^11^ ^]^ Most recently, Yan et al. reported that In_2_O_3_‐Pt/Al_2_O_3_ catalyst achieves ≈22% propylene yield at 450 °C with C_3_H_8_:O_2_ = 2:1 (32% propane conversion and 70% propylene selectivity).^[^ ^13^ ^]^ To the authors' knowledge, no ODHP process with O_2_ has been commercialized, mainly due to its low selectivity for propylene.^[^ ^14^ ^]^

Milder oxidants have also been investigated. CO_2_‐assisted ODHP (CO_2_‐ODHP, C_3_H_8_ + CO_2_ → C_3_H_6_ + CO + H_2_O) could, in theory, improve the thermodynamics of PDH.^[^ ^5^ ^]^ CO_2_ has two advantages: i) consumes H_2_ through the reverse water‐gas‐shift reaction (RWGS: CO_2_ + H_2_→ CO + H_2_O) increasing the equilibrium conversion of propane; ii) CO_2_ can remove coke via the reverse Boudouard reaction (CO_2_ + C→ 2CO).^[^ ^5^ ^]^ However, the presence of CO_2_ introduces the dry reforming of propane (C_3_H_8_ + 3CO_2_ → 6CO + 4H_2_) as a competing reaction.^[^ ^5^, ^15^, ^16^ ^]^ Although several key technical hurdles, such as selectivity control at high propane conversions and catalyst stability, have yet to be overcome,^[^ ^17^ ^]^ CO_2_‐ODHP is promising not only as a carbon‐negative technology in the context of the circular economy,^[^ ^5^ ^]^ but also provides a method of CO_2_ hydrogenation^[^ ^18^ ^]^ using the shale‐gas derived hydrogen.^[^ ^19^ ^]^ More catalyst and process development are needed to increase the propylene yield to make this approach commercially viable. There are several reviews that compare non‐oxidative PDH and ODPH processes,^[^ ^5^, ^8^, ^9^, ^16^, ^19^, ^20^, ^21^, ^22^ ^]^ and the rest of this article will focus on the catalyst development in non‐oxidative PDH.

### 1.2 Commercial Catalysts

Several PDH processes have been commercialized including Catofin, Olefex, STAR, PDH, ADHO, K‐PROTM, FBD‐4, and FCDh.^[^ ^9^ ^]^ Among these, the Catofin and the Oleflex processes are more widely deployed.^[^ ^23^ ^]^ The Catofin process employs fixed‐bed reactors with CrO*_x_*/Al_2_O_3_ catalysts containing more than 18% CrO*_x_* as the active ingredient and 1–2 wt% alkaline metals as promoters. It achieves propylene selectivities above 87% at 650 °C and 0.5 bar of propane. The Oleflex process employs fluidized bed reactors with Pt‐based catalysts, with propane conversion and propylene selectivity of 30–40% and 85.5–88%, respectively.^[^ ^8^, ^9^ ^]^ As synthesized CrO*_x_*‐based catalysts contain multiple oxidation states of Cr, including VI, IV, III, and II, but Cr^3+^ becomes the main species at reaction conditions.^[^ ^24^ ^]^ Coordinatively unsaturated Cr^3+^ has been proposed as the active site for PDH, with the reaction proceeding as follows: i) the adsorption of C_3_H_8_; ii) heterolytic dissociation of C—H bond to form a Cr‐C_3_H_7_ intermediate; iii) *β*‐H elimination of Cr‐C_3_H_7_ intermediate to form Cr^3+^‐C_3_H_6_ and H^−^ species; iv) propylene desorption and the formation of Cr‐H species; and v) desorption of H_2_ via proton transfer.^[^ ^5^ ^]^ The cleavage of the C—H bond of propane was proposed as the likely rate‐determining step (RDS).^[^ ^25^ ^]^ CrO*_x_*/Al_2_O_3_ catalysts deactivate quickly due to coke formation and aggregation of Cr^3+^ species, which can be mitigated using promoters such as Ce,^[^ ^26^ ^]^ Ni,^[^ ^27^ ^]^ and Zr.^[^ ^28^ ^]^

Pt‐based catalysts exhibit superior ability in activating C—H bonds in alkanes and low activity for C—C cracking and coke formation. Compared with other catalysts, Pt‐based catalysts show the highest turnover frequency (TOF) in PDH (**Table** **1**). TOF normalized by Pt, or TOF_Pt_, varies substantially with the support, the alloying metal, and the catalyst preparation method. The best‐reported TOF_Pt_ is more than 1000 times higher than that of TOF_Cr_ (Table 1). Propylene selectivity is high on Pt‐based catalysts (94–99%). The most accepted mechanism for Pt‐based catalysts is the Horiuti–Polanyi mechanism—first proposed in 1934—involving: i) cleavage of the first C—H bond in C_3_H_8_; ii) activation of the second C—H bond in C_3_H_7_; iii) formation of a dihydrogen molecule; iv) desorption of hydrogen and propylene.^[^ ^5^, ^29^ ^]^ The first C—H cleavage is considered the RDS.^[^ ^30^, ^31^ ^]^ Side reactions, such as hydrogenolysis, occur near the adsorbed hydrogen, which is favored on large Pt particles. Addition of a second metal to form bimetallic (intermetallic PtM, PtM alloy, or PtMO*_x_* clusters, M = metal) catalysts, such as PtSn,^[^ ^32^, ^33^, ^34^ ^]^ PtGa,^[^ ^35^, ^36^, ^37^, ^38^ ^]^ PtZn,^[^ ^30^, ^39^, ^40^, ^41^ ^]^ PtCu,^[^ ^42^ ^]^, and PtCo,^[^ ^43^ ^]^ can reduce the particle size of Pt, thereby improving the catalytic activity and inhibiting coke formation. Further information is available in these sources.^[^ ^5^, ^8^, ^14^, ^44^, ^45^, ^46^ ^]^

**Table 1:** Summary of recently reported catalysts for propane dehydrogenation

Sample	Temp. [°C]	Test conditions	Conv. [%]	Selectivity [%]	TOF [h^−1^]	Ref.
Pt‐based						
K‐PtSn@MFI	600	24% C_3_H_8_/He	38.7	>97	12 600	[47]
PtSn/Al_2_O_3_‐nanosheet	590	C_3_H_8_/H_2_/Ar = 1/0.5/2	48.7	99	5688	[48]
Pt‐Na/Sn‐ZSM‐5	590	C_3_H_8_/H_2_ = 3/1	41.7	≈95	792	[49]
PtZn/Al_2_O_3_	600	C_3_H_8_/H_2_/N_2_ = 1/1/2	35	94	4356	[50]
PtZn‐DeAlBEA	500	25.3% C_3_H_8_/He	18.2	>99	24 480	[51]
PtZn/SiO_2_	600	C_3_H_8_/H_2_ = 1/1	48	96	24 480	[30]
PtZn@Silicalite‐1	550	25% C_3_H_8_/N_2_	≈20	99.3	12 778	[41]
Ga^*δ*+^Pt^0^/SiO_2_	550	20% C_3_H_8_/Ar	31.9	>99	3348	[37]
Cr‐based						
CrO*_x_*/Al_2_O_3_	600	100% C_3_H_8_	33.2	90.4	20.4	[52]
Ce‐CrO*_x_*/Al_2_O_3_	630	14% C_3_H_8_/N_2_	86	78	0.75	[26]
Ni‐CrO*_x_*/Al_2_O_3_	550	10% C_3_H_8_/Ar	47	95	5.2	[27]
CrO_x_/H‐ZSM‐5	580	5% C_3_H_8_/N_2_	60.8	78.2	4	[53]
Ga‐based						
Ga_2_O_2_^2+^ (Ga/H‐ZSM‐5)	550	5% C_3_H_8_/N_2_	<10	91.6	2120	[54, 55]
Ga^+^ (Ga/H‐ZSM‐5)	550	5% C_3_H_8_/N_2_	<10	91.1	148	[54, 55]
Ga^+^ (Ga‐CHA)	550	5% C_3_H_8_/N_2_	<10	96	≈9	[56]
Ga/SiO_2_	550	20% C_3_H_8_/N_2_	9.3	94.3	20.4	[57]
Ga/Al_2_O_3_	620	5% C_3_H_8_/N_2_	46	95.2	42	[58]
Ga/SBA‐15	620	5% C_3_H_8_/N_2_	29.7	92	151	[58]
Ga‐MFI	600	5% C_3_H_8_/N_2_	12.2	82	34	[59]
Zn‐based						
Zn/H‐ZSM‐5	480	0.8% C_3_H_8_/He	<2%	88	8.8	[60]
ZnO/Silicalite‐1	550	5% C_3_H_8_/N_2_	49	≈90	7	[61]
ZnO*_x_*/Silicalite‐1	550	40% C_3_H_8_/N_2_	≈30	≈90	239	[62]
ZnO/DeAl‐Beta	600	5% C_3_H_8_/N_2_	≈50	93	5.8	[63]
V‐based						
VZrO_2_	550	13.3% C_3_H_8_/Ar	25	98	17	[64]
V/Si‐Beta	600	5% C_3_H_8_/N_2_	40	90	216	[65]
VO*_x_*/Al_2_O_3_	600	C_3_H_8_/H_2_/N_2_ = 1/1/1.6	15	94	16.2	[66]
Co‐based						
CoO/Silicalite‐1	600	5% C_3_H_8_/N_2_	40	94.7	30.26	[67]
Co/SiO_2_ (single atom)	550	23.8% C_3_H_8_/N_2_	25	95	196	[68]
Co‐Silicalite‐1	550	5% C_3_H_8_/N_2_	20	98.5	20	[69]
Co/*γ*‐Al_2_O_3_	590	C_3_H_8_/H_2_/N_2_ = 1/0.8/3.2	24.8	97.1	518	[70]
Co/Si‐Beta	600	5% C_3_H_8_/N_2_	40	96	32.9	[71]
Fe‐based						
FeP/Al_2_O_3_	600	5% C_3_H_8_/N_2_	15	>80	19	[72]
Fe/SiO_2_	650	3% C_3_H_8_/N_2_	<10	>99	1.1	[73]

Note: Conv., Selectivity, and TOF represent the propane conversion, propylene selectivity, and turnover frequency with mol_C3H8_ mol_Site_^−1^ h^−1^, respectively.

### 1.3 Metal‐Containing Zeolite Catalysts

**Figure 1:**
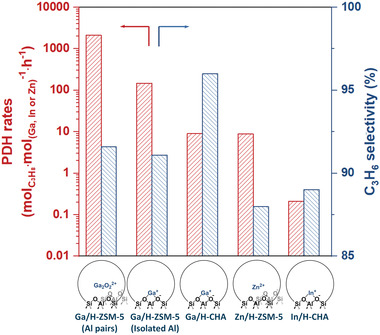
Comparison of PDH rates and C_3_H_6_ selectivity on different sites in metal/zeolites.

The high cost of Pt, as well as human health and environmental concerns associated with Cr, have incentivized the development of alternative PDH catalysts^[^ ^8^ ^]^ such as Ga‐,^[^ ^57^, ^74^, ^75^, ^76^, ^77^, ^78^, ^79^ ^]^ Zn‐,^[^ ^62^, ^80^, ^81^ ^]^ Co‐,^[^ ^70^, ^71^, ^82^ ^]^ V‐,^[^ ^66^, ^83^ ^]^ Fe‐,^[^ ^72^, ^84^, ^85^, ^86^ ^]^ Zr‐,^[^ ^87^, ^88^, ^89^ ^]^ Sn‐,^[^ ^90^ ^]^ and In‐based catalysts^[^ ^91^, ^92^ ^]^ and nanocarbon materials.^[^ ^93^ ^]^ Among these, single‐site (Ga^+^, Zn^2+^, Co^2+^, Fe^2+^, etc.) supported on silica or zeolites^[^ ^94^, ^95^, ^96^ ^]^ have shown particular promise. For Ga‐based catalysts, different supports, such as SiO_2_, Al_2_O_3_, SBA‐15, MFI, and H‐ZSM‐5, have been used to prepare Ga‐based catalysts, achieving TOF_Ga_ between 8 and 2120 h^−1^ (Table 1). Notably, Ga_2_O_2_^2+^ sites in H‐ZSM‐5 exhibit TOF_Ga_ at least one order of magnitude higher than other Ga‐based catalysts. Zn‐ and Co‐based catalysts show moderate levels of catalytic activity (TOF between 5 and 500 h^−1^), while Co‐related catalysts show a higher propylene selectivity (>95%) than Zn‐based catalysts (≈90%). V‐based catalysts show TOF between 16 and 216 h^−1^, and propylene selectivity between 90% and 98%. Fe‐based catalysts are slightly less active in comparison. Aside from the Pt‐based catalysts, Ga_2_O_2_^2+^ stabilized in H‐ZSM‐5 appears promising, achieving a significantly higher TOF than Ga^+^ in Ga/H‐ZSM‐5,^[^ ^54^, ^55^ ^]^ Zn^2+^ in Zn/H‐ZSM‐5,^[^ ^60^ ^]^ Ga^+^ in Ga‐CHA,^[^ ^56^ ^]^ and In^+^ in In‐CHA^[^ ^92^ ^]^ (**Figure** **1**). Although it is still more than ten times lower than that of Pt‐based catalysts, the marked difference in the cost of the two metals makes the Ga‐based catalyst a promising alternative.

**Figure 2:**
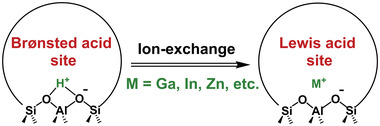
Schematic of the ion exchange process from Brønsted acid sites in H‐ZSM‐5 to Lewis acid site in metal cation‐exchanged zeolite catalysts.

Metal cation exchanged zeolite catalysts (Ga/H‐ZSM‐5, Zn/H‐ZSM‐5, Co/H‐ZSM‐5) are efficient catalysts for PDH with ≈90% propylene selectivity.^[^ ^54^, ^60^, ^97^ ^]^ As shown in **Figure** **2**, metal cations exchanged zeolites are prepared by replacing Brønsted acid sites (BAS) with metal cations via liquid phase ion‐exchange, reductive solid‐state ion‐exchange (RSSIE), or vapor‐phase ion‐exchange. Metal cations entering zeolite matrices via ion exchange (IE) generally exhibit Lewis acidity,^[^ ^98^ ^]^ and are generally believed to be responsible for catalyzing alkane dehydrogenation.^[^ ^76^, ^77^, ^99^ ^]^ However, identifying the exact structure of active metal species in the zeolite in PDH remains challenging.^[^ ^76^, ^77^, ^100^, ^101^, ^102^ ^]^

Ga‐based catalysts have a long history in hydrocarbon processing. Gallium oxide was investigated as a cracking catalyst as early as the 1960s.^[^ ^103^ ^]^ The dehydroaromatization activity of supported gallium oxide (on silica, alumina, and zeolites) was revealed in the patent literature in the 1970s,^[^ ^104^ ^]^ which led to the development of the Ga/H‐ZSM‐5 catalyst used in the CYCLAR process for converting propane and butane to aromatics.^[^ ^105^, ^106^, ^107^ ^]^ Since the dehydrogenation of hydrocarbons is the first step in dehydroaromatization, the ability of Ga/H‐ZSM‐5 to dehydrogenate light alkanes to alkenes was well appreciated. Meanwhile, the Ga speciation and the structure of active sites on Ga/H‐ZSM‐5 in dehydrogenation have remained active topics of discussion since its initial discovery without clear resolution. Different Ga species have been proposed to be the active center of the PDH, that is, Ga^+^, [GaO]^+^, [Ga(OH)_2_]^+^, [GaOH]^2+^, [GaH]^2+^, [GaH_2_]^+^, and Ga^+^−H^+^ pair.^[^ ^76^, ^77^, ^99^, ^100^, ^108^, ^109^ ^]^ Early studies suggested that Ga^+^ is the dominant species upon reduction of Ga/H‐ZSM‐5.^[^ ^99^ ^]^ However, recent investigations emphasized the importance of the framework Al pair site on the Ga speciation.^[^ ^76^, ^77^, ^100^ ^]^ We combined in situ transmission Fourier‐transform infrared (FTIR) spectroscopy, pulse titration studies, and PDH kinetics to demonstrate that the Ga speciation and their PDH performance are dependent on both Ga/Al ratios and Al distributions of the zeolites.^[^ ^54^, ^55^, ^110^ ^]^ The detailed discussion of Ga speciation and PDH mechanism will be included in the next section.

The ability of Zn/H‐ZSM‐5 to catalyze alkane dehydrogenation was recognized in the 1980s,^[^ ^111^ ^]^ and has been widely investigated ever since.^[^ ^81^, ^112^, ^113^, ^114^ ^]^ Similar to Ga/H‐ZSM‐5, multiple Zn species have been reported in Zn/H‐ZSM‐5, that is, isolated Zn^2+^, isolated [ZnOH]^+^, multinuclear ZnO clusters, and bulk ZnO aggregates.^[^ ^81^, ^112^, ^113^, ^114^ ^]^ Zn/H‐ZSM‐5 catalysts are typically prepared by the liquid IE method, which leads to the introduction of multi‐nuclear ZnO clusters and bulk ZnO, aside from the intended isolated Zn^2+^ sites.^[^ ^81^ ^]^ The fact that Ga^3+^ and Zn^2+^ are isoelectronic could play a role in their similarity in speciation when exchanged into zeolite pores and PDH activity. Almutairi et al.^[^ ^81^ ^]^ employed chemical vapor deposition (CVD) to prepare Zn/H‐ZSM‐5 with all BAS replaced by isolated Zn^2+^, which turned out to be less active for PDH than the samples prepared by IE and incipient wetness impregnation (IWI) methods. Gong et al.^[^ ^114^ ^]^ employed atomic layer deposition (ALD) to introduce ZnO to H‐ZSM‐5 and showed that isolated [ZnOH]^+^ was the dominant species after the first cycle of ALD. [ZnOH]^+^ had been proposed to be more active for PDH than bulky ZnO clusters formed after several ALD cycles. Nozik et al.^[^ ^60^ ^]^ recently employed solid‐state ion exchange of ZnCl_2_ with H‐ZSM‐5 to prepare Zn/H‐ZSM‐5 catalyst to investigate the effect of Zn/Al ratios on Zn speciation and PDH rates. Zn^2+^ was proposed to be the active site in the absence of H_2_. Those conflicting hypotheses could be caused by the different catalyst preparation methods (IE, IWI, CVD, ALD, or solid‐state ion exchange with ZnCl_2_), leading to the formation of different Zn species in Zn/H‐ZSM‐5. Bulk ZnO is known to heterolytically cleave H_2_ even at liquid nitrogen temperature,^[^ ^115^ ^]^ suggesting the possibility of the ZnO clusters in MFI pores to activate the H—H and C—H bond.

Indium‐based catalysts have also received attention for alkane dehydrogenation. Jones and co‐workers^[^ ^116^, ^117^ ^]^ investigated In_2_O_3_‐Ga_2_O_3_ and ternary In‐Ga‐Al mixed oxides as catalysts for PDH. It was found that In(0), formed during the reaction, was the cause of catalyst deactivation. Shimizu and co‐workers^[^ ^91^, ^118^ ^]^ first reported that In/H‐CHA was a stable and selective catalyst for ethane dehydrogenation. They also showed that the CHA zeolite was superior to other zeolite frameworks (BEA, MFI, and MOR) and that Al‐rich In/H‐CHA zeolite (Si/Al = 6.85) exhibited higher dehydrogenation rates. An isolated [InH_2_]^+^ site was proposed as the active center in these catalysts based on FTIR and X‐ray absorption fine structure (XAFS) measurements. The identification of [InH_2_]^+^ as the active site for ethane dehydrogenation was primarily based on ex situ FTIR spectroscopy and DFT calculations;^[^ ^91^, ^118^ ^]^ it should be noted that the spectra used in support of the presence of [InH_2_]^+^ were collected at low temperature (below 153 K, after treating with H_2_ at 773 K) rather than the reaction temperature. Lobo and co‐workers^[^ ^92^ ^]^ recently investigated the In speciation in In‐CHA catalysts through the combination of in situ FTIR spectroscopy, pulse titrations, and PDH kinetics. Different from the Ga‐CHA catalyst that Ga^+^ could react with H_2_ via oxidative addition to form GaH*_x_*, the InH*_x_* band was not observed on In‐CHA catalysts within a wide range of temperatures (50–550 °C), demonstrating that indium has a weaker metal‐hydrogen bond than gallium.^[^ ^56^ ^]^ It was concluded that isolated In^+^ sites rather than InH*_x_* were the stable active site for PDH, which was independent of the Al distribution. The low intrinsic PDH rate on In^+^ makes it less likely to be commercially relevant (Figure 1).

This review analyzes the mechanistic understanding of PDH on metal‐containing zeolite catalysts. We first focus on the characterization method and determination of active structure in Ga/H‐ZSM‐5 catalyst, as it is the most active in PDH in this category of catalysts. Then, we discussed the multiple PDH reaction mechanisms in Ga/H‐ZSM‐5. Finally, we provide perspectives on future directions to address unresolved questions and enable the development of more active and selective PDH catalysts.

## 2 Ga‐Containing Zeolites in PDH

Elucidating the Ga speciation on Ga/H‐ZSM‐5 at PDH conditions is a prerequisite for understanding the structure–activity relations. This is, however, far from trivial. PDH produces hydrogen, so the catalyst operates in a reducing environment (typically above 500 °C). Ga/H‐ZSM‐5 catalysts are commonly prepared by impregnating a Ga salt, for example, Ga(NO_3_)_3_, on the H‐ZSM‐5 support, followed by calcination in the air. At this point, Ga in the as‐synthesized Ga/H‐ZSM‐5 catalyst is typically at its highest oxidation state, that is, +3. Ga/H‐ZSM‐5 catalysts are then reduced in an H_2_ atmosphere at the reaction temperature before the feed is introduced. Thus, only characterizations conducted in situ/operando or at least on reduced Ga/H‐ZSM‐5 could provide relevant information regarding the speciation of Ga on the catalyst during PDH. The patent and academic literature in the 1970s and 1980s focused largely on the activity and product distribution,^[^ ^105^, ^106^, ^107^ ^]^ rather than the structure of the active center, which was likely due to the lack of in situ characterization with sufficient structural detail or spectral resolution.

### 2.1 Ga Speciation on Reduced Ga/H‐ZSM‐5

Understanding Ga speciation on Ga/H‐ZSM‐5 at PDH conditions is central to mechanistic understanding because the reaction pathway depends on the structure of the active site. PDH on Ga/H‐ZSM‐5 occurs at high temperatures (typically >500°C), and thus reaction intermediates are short‐lived and difficult to detect experimentally. Computational modeling plays a pivotal role in mapping out reaction pathways, and the reliability of the predicted pathways depends on the assumed catalytic structures. Investigations of Ga speciation on reduced Ga/H‐ZSM‐5 can be roughly divided into two periods. Earlier studies generally assumed that all intraporous Ga species in reduced Ga/H‐ZSM‐5 resided in a similar coordination environment, or different coordination environments did not impact PDH activity on the Ga species. Subsequent reports suggested that a few different Ga species, with distinct structures and PDH reactivities, were present in Ga/H‐ZSM‐5. In particular, the density of framework Al pairs in the zeolite framework is a key variable in determining the concentration of the most active Ga species in PDH. The following section addresses these two periods.

#### 2.1.1 Ga Speciation on Reduced Ga/H‐ZSM‐5 without Considering Al Distribution in Zeolite

Literature discussion of Ga speciation on Ga/H‐ZSM‐5 is fraught with conflicting claims, as conclusions are drawn based on results obtained with different characterization techniques. This is in part due to the fact that several key assumptions made in the data analysis in early studies were challenged by more recent experimental or computational evidence. Below, we discuss key information regarding the Ga speciation obtained from a number of techniques separately, before presenting an up‐to‐date understanding based on all existing evidence.

##### X‐ray Absorption Spectroscopy

X‐ray absorption spectroscopy (XAS) could be conducted in situ or operando, and thus is well suited for determining the Ga speciation in Ga/H‐ZSM‐5 at PDH conditions. Meitzner et al. first employed in situ Ga K edge XAS to investigate the reduction of Ga/H‐ZSM‐5 catalysts.^[^ ^99^ ^]^ As synthesized Ga/H‐ZSM‐5 catalyst was prepared by impregnation and calcination. Four standard samples were employed in the interpretation of the spectra collected on Ga/H‐ZSM‐5:

1. Ga metal;
2. framework Ga‐ZSM‐5, in which Ga isomorphously substituted Si in the zeolite framework in the tetrahedral coordination configuration;
3. Ga(NO_3_)_3_ in which Ga is in the octahedral coordination environment;
4. fully reduced Ga/H‐ZSM‐5 by H_2_ at 507 °C (attributed to GaH*_x_*).

**Figure 3:**
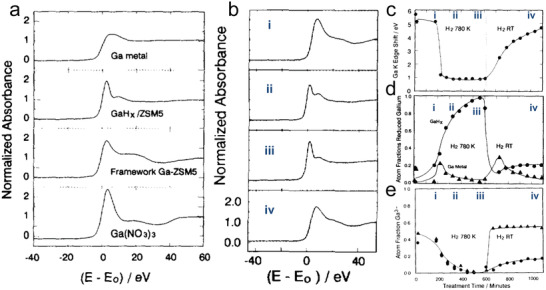
a) Near‐edge XAS spectra of four standards. GaH*_x_*/ZSM‐5 represented the status of reducing Ga/H‐ZSM‐5 (14.5, 0.27) at 507 °C for 6–8 h. b) Near‐edge XAS spectra of Ga/H‐ZSM‐5 (14.5, 0.27) at different stages of catalyst treatment: i) at the beginning of H_2_ treatment at 507 °C; ii) at the intermediate state of reduction; iii) after 6–8 h of reduction; iv) cooled down to room temperature in the presence of H_2_ after reduction. c) Ga K edge energy shifts, d) atomic fraction of Ga species, e) atomic fraction of Ga^3+^ species during reduction and cooling, as described in (b). Triangles and circles in (d) represent the Ga metal and GaH*_x_*, respectively. Triangles and circles in (e) represent the tetrahedral Ga^3+^ and octahedral Ga^3+^, respectively. a‐e) Adapted with permission.^[^ ^99^ ^]^ Copyright 1993, Elsevier.

Spectral assignments were made based on the distinct Ga K edge energies for these species, though later results showed that the edge energy could not be treated as diagnostic information regarding the Ga species at different oxidation states (see below).^[^ ^119^ ^]^ The XAS spectra of all standard samples and their Ga K edge energy (*E*) and Δ*E* (referenced to the first inflection point in the edge region, **Figure** **3**a and **Table** **2**). During the treatment of the catalyst in H_2_ at 507 °C, the Ga K edge decreased in energy (Figure 3b–e), suggesting the reduction of Ga species in the as‐synthesized sample. The decrease of Ga K edge energy shifts of Ga/H‐ZSM‐5 during the reduction was confirmed by several research groups.^[^ ^77^, ^100^, ^108^, ^119^, ^120^ ^]^ This transition was attributed to the conversion of Ga^3+^ species to GaH*_x_* by Meitzner et al.^[^ ^99^ ^]^ The amount of H_2_ consumed during the reduction of the as‐synthesized Ga/H‐ZSM‐5 was consistent with the reduction of Ga(III) to Ga(I). Interestingly, the Ga K edge of the reduced sample increased as the reduced sample was cooling down to room temperature in H_2_, a trend attributed to the oxidation of GaH*_x_* to Ga^3+^ species in an extraframework tetrahedral coordination environment (Figure 3b–e). The oxidizing agent responsible for the oxidation of GaH*_x_* was speculated to be protons.^[^ ^99^ ^]^

**Table 2:** Summary of absolute energies of Ga K absorption edge for Ga‐related species. Adapted with permission.^[^ ^99^ ^]^ Copyright 1993, Elsevier

Sample	Edge energy [eV]	Δ*E* [eV]
Ga metal	10 367.1	0
GaH*_x_* in H‐ZSM‐5	10 368.0	0.9
^a)^Tetrahedral Ga^3+^	10 372.7	5.6
^b)^Octahedral Ga^3+^	10 373.3	6.2

^a)^ from framework Ga‐ZSM‐5;

^b)^ from Ga(NO_3_)_3_.

The interpretation of Ga K edge energy shift has evolved as new experimental evidence is accumulated. Hensen and co‐workers attributed this observation to the reduction of Ga^3+^ to Ga^+^,^[^ ^108^ ^]^ a conclusion supported by Faro's and Lercher's investigations.^[^ ^77^, ^120^ ^]^ The assignment of the reduced Ga species in Ga/H‐ZSM‐5 to Ga^+^ and GaH*_x_* was often made based on similar XAS results, reflecting distinct mechanistic hypotheses by different researchers. In an important work, Hock and co‐workers compared the X‐ray absorption near edge spectroscopy (XANES) spectra of a series of model organometallic Ga compounds with well‐defined structures and concluded XANE spectra could not provide diagnostic information regarding the oxidation state of Ga.^[^ ^119^ ^]^ The identity and number of ligands coordinating with Ga could give rise to changes in the XANE spectra similar to those expected of changes in the oxidation state of Ga. Thus, the shift of Ga K edge energy to lower energy upon exposure to H_2_ or propane could be attributed to several causes, that is, conversion of Ga^3+^ to i) Ga^+^, 2) GaH*_x_*, and 3) Ga^3+^ coordinated with alkyl groups. Bell and co‐workers attributed the ≈4.6 eV decrease in the Ga K edge energy upon switching from O_2_ to H_2_ atmosphere of Ga/H‐ZSM‐5 at or above 500 °C to the transformation of [Ga(OH)_2_]^+^−H^+^ to [GaH]^2+^.^[^ ^100^ ^]^ Thus, edge shifts in XAS alone cannot unambiguously determine the oxidation state of Ga during the PDH.

In situ XAS investigations provided crucial information regarding how Ga species migrate into the zeolite pore upon reduction. Ga exists mostly in the form of Ga_2_O_3_ on the external surface of H‐ZSM‐5 in as‐synthesized Ga/H‐ZSM‐5, evidenced by the equal distribution of Ga in the tetrahedral and the octahedral coordination environments.^[^ ^99^ ^]^ Ga species are mobilized upon reduction or coordination with H or alkyl groups at high temperatures (>500 °C), before entering into the zeolite pore. Oxidation of reduced Ga/H‐ZSM‐5 leads to the formation of mostly tetrahedrally coordinated Ga^3+^ species, suggesting that they are located in the zeolite pore rather than in the form of Ga_2_O_3_ crystallites.^[^ ^99^ ^]^ Reduction of intraporous Ga^3+^ species is more facile than those in Ga_2_O_3_ on the surface of zeolite because no Ga migration is needed.

##### In Situ FTIR Spectroscopy

**Figure 4:**
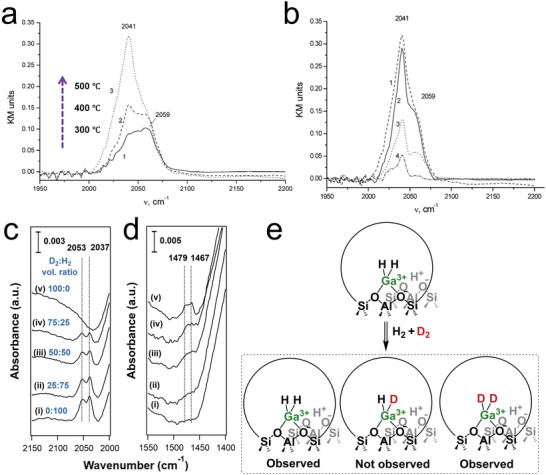
a) DRIFT spectra of gallium hydrides resulting from hydrogen adsorption (6.7 kPa H_2_) at different temperatures on reduced Ga/HZSM‐5 (reduced at 500 °C and evacuated at 600 °C): 1) 300 °C; 2) 400 °C, and 3) 500 °C. b) DRIFT spectrum of decomposition of gallium hydrides upon heating in vacuum at elevated temperatures: 1) the sample was reduced in hydrogen at 500 °C and then cooled in hydrogen to room temperature, 2) the same sample after evacuation at 300 °C, 3) evacuation at 400 °C, and 4) evacuation at 500 °C. a,b) Adapted with permission.^[^ ^109^ ^]^ Copyright 2004, Elsevier. c,d) Transmission FTIR spectra of Ga/H‐ZSM‐5 at 450 °C in c) high and d) low wavenumber ranges. The spectra were collected in the presence of a mixture of D_2_ and H_2_, where D_2_ and H_2_ have different ratios (indicated in the figure legends), and the total pressure is 1 atm. The sample was reduced at 550 °C, followed by cooling to 450 °C under vacuum. e) H‐D exchange mechanism of [GaH_2_]^+^ in a mixture of H_2_ and D_2_. c‐e) Adapted with permission.^[^ ^54^ ^]^ Copyright 2022, American Chemical Society.

The chemical specificity of FTIR spectroscopy makes it well‐suited for identifying reaction intermediates. Hensen and co‐workers^[^ ^109^, ^121^ ^]^ applied diffuse reflectance infrared Fourier Transform spectroscopy (DRIFTS) to the reduction of Ga/H‐ZSM‐5 and reported the first definitive spectral evidence of Ga hydrides on reduced Ga/H‐ZSM‐5. Upon reduction at 500 °C, the intensity of BAS OH groups at 3610 cm^−1^ decreased gradually with time, because of the replacement of BAS OH groups by Ga species. The decrease of the BAS band at 3610 cm^−1^ during hydrogen treatment of Ga/H‐ZSM‐5 at 500 °C over time, combined with the knowledge that Ga species enter into the zeolite pore during reduction, is compelling evidence that cationic Ga species replaced protons as the charge balancing species after entering the zeolite pore. A 1:1 correlation between the amount of Ga entering the zeolite pore and that of BAS consumed was later obtained with quantitative in situ transmission FTIR spectroscopy with pyridine as the probe molecule on reduced Ga/H‐ZSM‐5 samples.^[^ ^110^ ^]^ Exposure of reduced Ga/H‐ZSM‐5 to H_2_ at a temperature above 300 °C led to the formation of bands at 2041 and 2059 cm^−1^ (**Figure** **4**a) in the Ga hydride spectral region (1900–2100 cm^−1^)^[^ ^122^ ^]^. The Ga hydride band intensity decreases at temperatures above 300 °C, and higher temperatures led to near‐complete Ga hydride decomposition, with the peak at 2059 cm^−1^ disappearing first (Figure 4b). The band at 2041 cm^−1^ was assigned to the Ga dihydride ([GaH_2_]^+^), while the 2059 cm^−1^ band was attributed to the Ga‐H mode in [Ga^3+^(OH^−^)(H^−^)],^[^ ^109^, ^121^ ^]^ typically referred to as Ga monohydride in the later literature.

It should be noted that these assignments to specific Ga hydride species were largely speculative, but were widely cited. The proposed [GaH_2_]^+^ species contains two equivalent H so that if Ga/H‐ZSM‐5 is reduced in an H_2_/D_2_ mixture, a binomial mixture of [GaH_2_]^+^, [GaHD]^+^, [GaD_2_]^+^ would be expected. When H_2_/D_2_ mixtures with different compositions were employed as the reducing atmosphere in a recent study, only Ga‐H and Ga‐D modes were observed (Figure 4c,d). The absence of a distinct band for [GaHD]^+^ calls the existence of such a species into question (Figure 4e). It is likely that both the 2041 and 2059 cm^−1^ bands correspond to a Ga monohydride species located in slightly different environments in the zeolite pore (we will return to this point later).

#### 2.1.2 Impact of Al Distribution in H‐ZSM‐5 on the Ga Speciation

A frequent implicit assumption of the reports discussed above is that all intraporous Ga species have a similar structure and PDH activity; thus, the characterization results obtained reflected the properties of all active Ga species in PDH. Since PDH activity increases significantly (more than one order of magnitude) when Ga is introduced to H‐ZSM‐5, the intraporous Ga species is presumed to be the main active site. However, this assumption implies that the TOF should be independent of Ga loading and the Si/Al ratio of the H‐ZSM‐5 support, as long as most Ga enters into the zeolite and sufficient BAS are present: these predictions turned out to be wide off the mark.

##### Impact of the Ga/Al Ratio on the Ga Speciation

**Figure 5:**
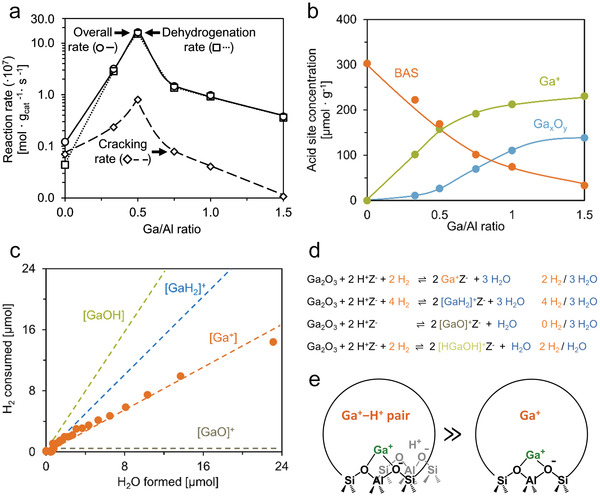
a) PDH and cracking rates as a function of the Ga/Al ratio of the Ga/H‐ZSM‐5 catalysts (Si/Al = 50, reaction temperature 510 °C, propane partial pressure 8.75 kPa). b) LAS and BAS concentrations of the reduced Ga/H‐ZSM‐5 catalysts as a function of the Ga/Al ratio. c) H_2_ consumption as a function of H_2_O formation during the pulsed TPR of the Ga/H‐ZSM‐5 (50, 0.5) catalyst. d) Possible reactions between H_2_ and Ga_2_O_3_ during the reduction of Ga/H‐ZSM‐5 with distinct stoichiometries. e) Proposed active site for PDH. a‐e) Adapted with permission.^[^ ^77^ ^]^ Copyright 2018, American Chemical Society.

Lercher and co‐workers^[^ ^77^ ^]^ were the first to examine the impact of the Ga/Al ratio on Ga speciation and PDH rates: they observed that the rate increased as Ga/Al ratio rises from 0 to 0.5, before declining with further addition of Ga (**Figure** **5**a). A similar trend was observed for the propane cracking reaction (C_3_H_8_ → CH_4_ + C_2_H_4_), indicating that both PDH and cracking occur on the same active site (a similar trend was later reported by Yuan et al.).^[^ ^55^ ^]^ The non‐monotonic trend of the PDH rate with increasing Ga loading suggested that a single type of active Ga species dominating PDH activity over the entire range of Ga/Al ratio was unlikely. If there were only one type of active Ga species for PDH in Ga/H‐ZSM‐5, then the density of this species was expected to increase with Ga loading until the thermodynamic driving force for additional Ga to enter the zeolite was exhausted, which would lead to leveling off, rather than falling of PDH activity. Importantly, Ga species in reduced Ga/H‐ZSM‐5 with different Ga loadings were determined quantitatively. A 1:1 exchange ratio between Ga and BAS was determined at Ga/Al ratios below 0.5, a result confirmed later by Yuan et al.^[^ ^110^ ^]^

It was also found that at Ga/Al ratios above 0.5, Lewis acidic Ga*_x_* O*_y_* species started to appear (Figure 5b). In this report,^[^ ^77^ ^]^ H_2_‐pulsed temperature‐programmed reduction (TPR) was applied to quantitatively determine the amounts of H_2_O formed and H_2_ consumed during the reduction of Ga/H‐ZSM‐5 (Figure 5c). Comparison of the measured H_2_/H_2_O ratio of ^2^/_3_ with the stoichiometry of potential reactions points to Ga^+^ as the dominant Ga species formed upon reduction of Ga/H‐ZSM‐5 (Figure 5d). To reconcile with the observed TOF trends at different Ga/Al ratios, a Lewis–Brønsted acid pair site (Ga^+^−H^+^ pair) was proposed as a highly active species in PDH based primarily on the results of DFT calculations (Figure 5e). A synergistic effect between neighboring Ga^+^ and H^+^ sites was envisioned, leading to the optimal PDH activity at an equal density of the two types of sites. The proximity of Ga^+^ and H^+^ in the proposed mechanism suggested that the Ga^+^−H^+^ pair was only present when two framework Al atoms were located close to each other. This was the first time that the distribution of framework Al atoms in zeolites was considered, if not explicitly, as a relevant variable in PDH on Ga/H‐ZSM‐5. Deng and co‐workers also confirmed the synergy between BAS and Ga cations species in Ga/H‐ZSM‐5 zeolites in the methanol‐to‐aromatics reaction.^[^ ^123^ ^]^ Solid state NMR spectroscopy has been employed to provide evidence for the migration of Ga species into zeolites pores, and importantly, to estimate the distances of BAS pairs and Ga‐H pairs through ^1^H−^1^H SQ‐DQ and ^1^H−^71^Ga S‐RESPDOR, respectively.^[^ ^123^, ^124^, ^125^ ^]^ It should be noted that the measured Ga/H‐ZSM‐5 is in the oxidized, rather than reduced, form.

**Figure 6:**
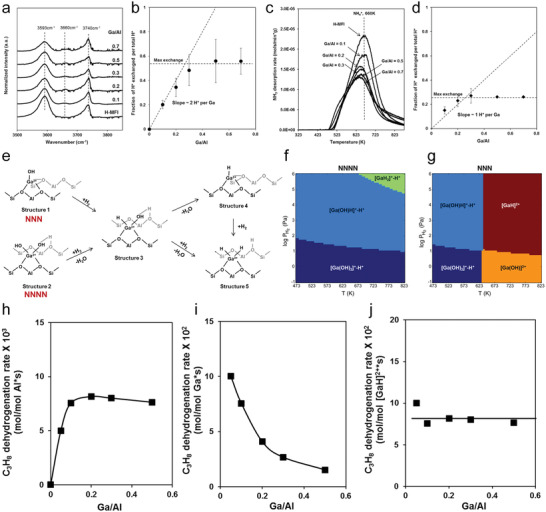
a) Infrared spectra of H‐ZSM‐5 and Ga/H‐ZSM‐5 with Ga/Al ratio ranging from 0.1 to 0.7 at 450 °C under flowing dry air. b) Fraction of BAS O—H groups (3593 cm^−1^ in (a)) as a function of Ga/Al ratio. Dotted lines indicate the slope of the plot and the maximum extent of H^+^ exchange. c) NH_3_‐TPD profiles of H‐ZSM‐5 (16.5) and Ga/H‐ZSM‐5 (16.5, Y). d) Fraction of BAS exchanged, determined from NH_3_‐TPD profiles, as a function of Ga/Al ratio. Dotted lines indicate the slope of the plot and the maximum extent of BAS. e) Pathways for reduction of [Ga(OH)]^2+^ cations and [Ga(OH)_2_]^+^‐H^+^ cation pairs. f,g) Theoretical thermodynamic phase diagrams for Ga^3+^ structures at cation‐exchange sites associated with f) NNNN and g) NNN proximate framework Al atoms. Colored regions reflect a Ga^3+^ structure that has the lowest free energy of formation from [Ga(OH)_2_]^+^‐H^+^ cation pairs at a corresponding temperature (*T*) and hydrogen partial pressure (*P*_H2_) and water partial pressure (*P*_H2O_) of 10 Pa. The H_2_O partial pressure used for this diagram is representative of conditions prevalent during H_2_‐TPR and NH_3_‐TPD. a‐g) Adapted with permission.^[^ ^100^ ^]^ Copyright 2018, American Chemical Society. h–j) C_3_H_8_ dehydrogenation rates as a function of Ga/Al ratio: h) Rates normalized per Al atom, i) rates normalized per Ga atom, and j) rates normalized per [GaH]^2+^ estimated via NH_3_‐TPD. Reaction conditions: 0.9 kPa C_3_H_8_/He and 460 °C. h‐j) Adapted with permission.^[^ ^76^ ^]^ Copyright 2019, American Chemical Society.

Bell and co‐workers recognized the importance of the framework Al distribution in PDH more explicitly.^[^ ^76^, ^100^ ^]^ Ga/H‐ZSM‐5 catalysts were prepared via vapor‐phase exchange of H^+^ with GaCl_3_ at 205 °C, followed by H_2_ reduction at 550 °C to remove Cl ligands. The resulting material was then oxidized by O_2_ at 500 °C for further characterization. Infrared spectra of Ga/H‐ZSM‐5 (under oxidizing conditions at 450 °C) showed that the intensity of the BAS OH group (3593 cm^−1^) decreased monotonically as the Ga/Al ratio rises to 0.3, a result of the replacement of BAS by Ga^3+^ species (**Figure** **6**a). The appearance of an IR band at 3660 cm^−1^ was assigned to the Ga‐OH band. The integration of the band at (3593 cm^−1^) was used for the quantification of BAS consumption as a function of Ga/Al ratios (Figure 6b). At Ga/Al ratio below 0.3, each Ga^3+^ replaces approximately two Brønsted acid O—H groups, consistent with the formation of divalent [Ga(OH)]^2+^ cations. For Ga/Al ratios higher than 0.3, the BAS was not exchanged further, a result assigned to the formation of GaO*_x_* oligomers that do not occupy cation‐exchanged sites. NH_3_ temperature‐programmed desorption (NH_3_‐TPD) experiments were conducted to further probe the exchange between Ga^3+^ sites and BAS on oxidized Ga/H‐ZSM‐5 (Figure 6c,d). The NH_3_ desorption peak at ≈387 °C was attributed to the desorption of NH_3_ interacting with BAS O—H groups, and the integrated peak area used to estimate the concentration of BAS in Ga/H‐ZSM‐5 catalysts.

For Ga/Al ratios below 0.3, NH_3_‐TPD results showed that each Ga^3+^ site titrated approximately one BAS (Figure 6d), which is inconsistent with the analysis of infrared spectra (Figure 6a,b). The authors proposed the presence of two types of Ga sites associated with paired framework Al atoms: i) [GaOH]^2+^ exchanged with two protons that were next‐nearest neighbors (NNN), and ii) [Ga(OH)_2_]^+^ exchanged with a proton with another proton as the next‐next‐nearest neighbor (NNNN), as in Figure 6e. NNN and NNNN were calculated to be the paired Al framework atoms with a different distance between the two Al atoms (less than 5 Å and more than 5Å, respectively). The different conclusions drawn from infrared spectroscopy and NH_3_‐TPD were reconciled based on the different detection mechanisms of the two techniques. [GaOH]^2+^ exchanged with NNN exhibited the same exchange stoichiometry in both techniques that one Ga atom replaces two BAS sites. In [Ga(OH)_2_]^+^−H^+^ the O—H stretching mode of the BAS was shifted from 3610 to 2436 cm^−1^ due to the H‐bonding with the neighboring Ga‐OH group, leading to the apparent exchange stoichiometry of one Ga atom for two BAS based on the FTIR. Meanwhile, the BAS in [Ga(OH)_2_]^+^−H^+^ could still be detected by NH_3_‐TPD. Subsequent reduction of [GaOH]^2+^ and [Ga(OH)_2_]^+^−H^+^ led to the formation of [HGaOH]^+^−H^+^, and then [GaH]^2+^ and [GaH_2_]^+^−H^+^, respectively (Figure 6e).

Thermodynamic phase analysis (Figure 6f,g) showed that the Ga^3+^ structure was dependent on both H_2_ pressure and temperature: the Ga‐OH group in both [GaOH]^2+^ and [Ga(OH)_2_]^+^−H^+^ sites are stable at either lower temperature or low H_2_ pressure. High H_2_ pressure and high‐temperature lead to the formation of corresponding GaH*_x_* species, which were regarded as stable species upon reduction of Ga/H‐ZSM‐5 catalysts at high temperatures. The increase in PDH rate with Ga/Al ratios (Figure 6h) showed that PDH rates increase as Ga/Al ratios rise to 0.1 and level off with the further increase in Ga/Al ratios. Ga normalized PDH rates showed a decreasing catalytic rate with the increase of Ga loading (Figure 6i), indicating the highly active Ga species were introduced at low Ga loading, and a higher Ga loading led to the formation of less active or inactive Ga species. The [GaH]^2+^ (estimated via NH_3_‐TPD) normalized reaction rate was independent of the Ga/Al ratio (Figure 6j), based on which [GaH]^2+^ was assigned as the active center in PDH.

Schreiber et al. and Phadke et al.^[^ ^76^, ^77^ ^]^ both pointed out the importance of framework Al pairs in the formation of active species for PDH, however, they proposed different active site structures and reaction mechanisms. Here, we discuss possible causes leading them to different conclusions. Ga/H‐ZSM‐5 catalysts were prepared via different methods, that is, conventional impregnation and vapor‐phase exchange of H^+^ with GaCl_3_. The vapor‐phase exchange approach could ensure Ga species were only introduced via the ion‐exchange mechanism. On samples prepared by impregnation at Ga/Al ratios ≤ 0.5, a 1:1 exchange ratio between Ga and BAS was determined after reduction at high temperature with hydrogen, suggesting complete conversion of Ga_2_O_3_ into intraporous cationic Ga species. Thus, Ga species are expected to exist only the in cationic form balancing negative charges of the framework in reduced Ga/H‐ZSM‐5 samples (Ga/Al ≤ 0.5), regardless of the preparation methods. The potential for preferential siting of cationic Ga species with different synthesis methods, however, cannot be ruled out, although it has not been substantiated by experimental evidence.

Another key difference is the state of catalysts when the densities of BAS were determined. While the BAS density was determined on reduced Ga/H‐ZSM‐5 by Schreiber et al., Phadke et al. conducted both FTIR and NH_3_‐TPD on the oxidized form of catalysts. The proposed mechanism by Phadke et al. (Figure 6e) suggested that the density of BAS remained unchanged regardless of the oxidation state of the catalyst.^[^ ^100^ ^]^ The opposite was also reported, where BAS density increased upon oxidation of reduced Ga/H‐ZSM‐5.^[^ ^110^, ^121^ ^]^ In addition, quantification of BAS density using pyridine as a probe molecule is more accurate than the estimate based on the area of the O—H band (≈3600 cm^−1^) because this band is easily obscured by the background at low BAS densities.^[^ ^126^ ^]^ This is relevant because densities of active Ga species are typically calculated based on measured BAS densities. Schreiber et al. also claimed that Ga mostly existed in the form of Ga^+^ in reduced Ga/H‐ZSM‐5 based on the H_2_ pulse reaction analysis, while the presence of Ga^+^ was not considered by Phadke et al. Subsequent reports from the same group^[^ ^76^, ^101^ ^]^ found Ga species exchanged with paired framework Al atoms and isolated framework Al atoms exhibited similar catalytic properties in ethane dehydrogenation. It should also be noted that Schreiber et al. and Phadke et al. employed Ga/H‐ZSM‐5 with quite different Si/Al ratios of the zeolite support (50 and 16.5, respectively). Assuming Al is distributed randomly in the zeolite framework, Al‐rich zeolites would have higher densities of paired framework Al sites, which could have a sizable impact on Ga speciation.

##### Impact of Ga/Al and Si/Al Ratios on Ga Speciation

**Figure 7:**
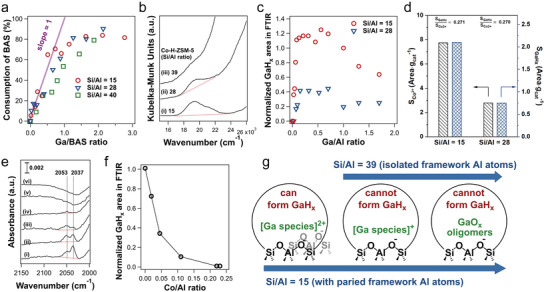
a) BAS consumption determined by quantitative infrared spectroscopy as a function of Ga/BAS ratios on Ga/H‐ZSM‐5 samples with varying Si/Al ratios (15, 28, and 39). b) UV–vis spectra of Co‐H‐ZSM‐5 with varying Si/Al ratios (15, 28, and 39). The red dotted line represents the baseline for the integration. c) Integrated GaH*_x_* peak (both 2037 and 2053 cm^−1^) area determined via quantitative infrared spectroscopy (normalized to the GaH*_x_* peak area of Ga/H‐ZSM‐5 (15, 1.0)) as a function of the Ga/Al ratio. d) *S*_Co2+_ (peak area of Co^2+^ normalized by a per gram of catalyst) and *S*_GaH_ *_x_* (maximized GaH*_x_* peak area normalized by a per gram of catalyst) between the Si/Al ratio of 15 and 28 with Ga/Al ratios of 0.13 and 0.15, respectively. e) FTIR spectra of H_2_‐treated Ga/Co‐H‐ZSM‐5 (15, 0.3, Z) with different Co/Al ratios collected at 550 °C: i) Co/Al = 0; ii) Co/Al = 0.019; iii) Co/Al = 0.045; iv) Co/Al = 0.11; v) Co/Al = 0.22; and vi) Co/Al = 0.23. f) Integrated GaH*_x_* peak area determined via quantitative FTIR spectroscopy as a function of the Co/Al ratio (the peak area was normalized to the GaH*_x_* peak area of Ga/Co‐H‐ZSM‐5 (15, 0.3, 0)). g) Exchanged Ga species and their ability to form GaH*_x_* at 550 °C on reduced Ga/H‐ZSM‐5 with Si/Al ratios of 15 and 39 with the incremental Ga/Al ratios. a,c) Adapted with permission.^[^ ^110^ ^]^ Copyright 2021, Elsevier. b,d‐g) Adapted with permission.^[^ ^54^ ^]^ Copyright 2022, American Chemical Society.

To gain a more thorough picture of Ga speciation, it is important to systematically investigate Ga/H‐ZSM‐5 samples with different densities of framework Al pairs and Ga loadings. In general, lower Si/Al ratios are expected to lead to higher densities of framework Al pairs in zeolites. Yuan et al. investigated Ga/H‐ZSM‐5 samples with three Si/Al ratios of the zeolite support, that is, 15, 28, and 39, and each in a wide range of Ga loadings.^[^ ^54^, ^55^, ^110^ ^]^ The notation of Ga/H‐ZSM‐5 (X, Y) is employed below, with X and Y denoting the Si/Al ratio and the Ga/Al ratio, respectively. As mentioned above, a 1:1 Ga/BAS exchange ratio was observed on reduced Ga/H‐ZSM‐5 with all three Si/Al ratios at sufficiently low Ga/Al ratio (**Figure** **7**a). The Ga/BAS exchange ratio started to deviate from unity at Ga/BAS ratios of ≈0.7, ≈0.5, and ≈0.4 on Ga/H‐ZSM‐5 (15, Y), Ga/H‐ZSM‐5 (28, Y), and Ga/H‐ZSM‐5 (39, Y), respectively, suggesting that not all Ga existed as cationic species. BAS cannot be completely exchanged by Ga at Ga/Al ratios as high as 1.7. This is an indication that the formation of Ga oligomers in the zeolite micropores is more favored as BAS becomes scarce,^[^ ^100^ ^]^ and that a fraction of BAS could be located at crystallographic positions inaccessible to Ga species.

The density of framework Al pairs can be titrated by Co^2+^ cations because only two adjacent framework negative charges can balance the divalent cation.^[^ ^127^, ^128^ ^]^ As expected, the amount of exchanged Co^2+^ decreased for increasing Si/Al ratios of the zeolites (Figure 7b), with the Co^2+^ exchanged in H‐ZSM‐5 (39) barely above the detection limit. On reduced Ga/H‐ZSM‐5(39, Y) at 550 °C, the GaH*_x_* band (2037 and 2053 cm^−1^) was absent regardless of the Ga/Al ratio, supporting the correlation between GaH*_x_* and framework Al pairs. The GaH*_x_* band initially increased in intensity with Ga loading before leveling off at the Ga/Al ratio of 0.1 on reduced Ga/H‐ZSM‐5 (15, Y), and declined as the Ga/Al ratio rose above 0.56 (Figure 7c). A similar observation was made on Ga/H‐ZSM‐5 (28, Y), but with a lower maximum GaH*_x_* band intensity than that of Ga/H‐ZSM‐5 (15, Y). In contrast, the consumption of the BAS by Ga exchange upon reduction did not stop until the Ga/Al ratio exceeds 0.7 in both samples (Figure 7a). These diverging trends in the formation of GaH*_x_* and the consumption of BAS show that not all cationic Ga species are capable of forming Ga hydrides upon reduction, which is also consistent with the lack of GaH*_x_* on Ga/H‐ZSM‐5 (39, Y). Ratios between the maximum integrated area of the GaH*_x_* peak and the UV–vis band corresponding to Co^2+^ are identical within experimental errors on Ga/H‐ZSM‐5(15, Y) and Ga/H‐ZSM‐5 (28, Y) (Figure 7d), displaying a linear correlation between the framework Al pair density and the amount of GaH*_x_*. When Ga was supported on (partially) Co^2+^ exchanged H‐ZSM‐5 (15), the intensity of the GaH*_x_* band was inversely correlated with the amount of Co^2+^ exchanged in the zeolite (Figure 7e,f), further supporting this connection. Since only framework Al pairs can stabilize divalent cationic species, the correlation between densities of framework Al pairs and GaH*_x_* species indicates that the precursor of GaH*_x_* is likely a divalent cationic species (Figure 7g).

**Figure 8:**
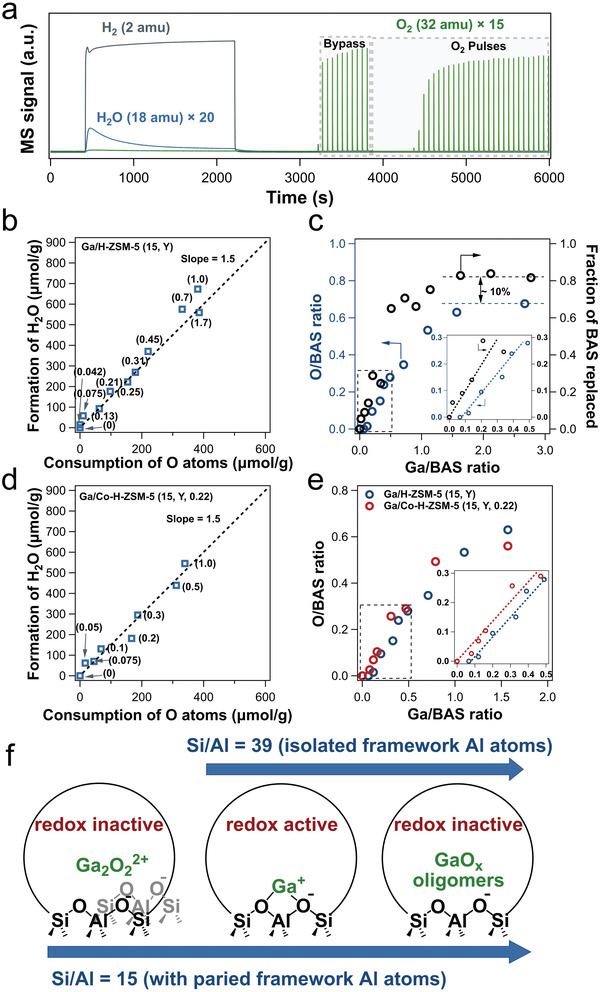
a) H_2_, H_2_O, and O_2_ MS signals versus time during the reduction and reoxidation of Ga/H‐ZSM‐5 (15, 1.7). The reduction was conducted in a 10 vol% H_2_ flow at 550 °C and subsequently oxidized by introducing pulses containing 10 vol% O_2_ to the catalyst bed at 550 °C. b) Relationship between the amount of H_2_O formed and O atoms consumed on Ga/H‐ZSM‐5 (15, Y) catalysts was investigated (the corresponding Ga/Al ratio is shown in parentheses). c) O/BAS ratio and the fraction of BAS replaced by Ga during reduction as a function of the Ga/BAS ratio of catalysts (inset replots the data in the low Ga/BAS range of (c)). d) Relation of the amount of H_2_O formed and O atoms consumed for Ga/Co‐H‐ZSM‐5 catalysts investigated (the corresponding Ga/Al ratio is shown in parentheses). e) O/BAS of Ga/H‐ZSM‐5 (15, Y) and Ga/Co‐H‐ZSM‐5 (15, Y, 0.22) as a function of the Ga/BAS ratio. f) Exchanged Ga species and their redox properties at 550 °C on reduced Ga/H‐ZSM‐5 with Si/Al ratios of 15 and 39 with the incremental Ga/Al ratios. Arrows indicate increasing Ga loading. a‐f) Adapted with permission.^[^ ^54^ ^]^ Copyright 2022, American Chemical Society.

The composition of the divalent cationic Ga species was deducted by the amount of oxygen needed to fully oxidize reduced Ga/H‐ZSM‐5 via a quantitative pulse titration method.^[^ ^54^ ^]^ In a typical experiment (**Figure** **8**a), the reduction of as‐synthesized Ga/H‐ZSM‐5 (15, 1.7) with 10 vol% H_2_/N_2_ at 550 °C led to significant H_2_O formation, evidencing the reduction of the supported Ga_2_O_3_, and the subsequent O_2_ pulses oxidizing reduced Ga species. Determination of the amounts of H_2_O formed during the reduction and O_2_ consumed in the subsequent oxidation provide direct evidence for the change in the oxidation state of Ga species in the reduced and oxidized sample. The molar ratio between water formed in the reduction, and oxygen consumed in Ga/H‐ZSM‐5 (15, Y) samples (Y = 0–1.7) was ≈1.5 (Figure 8b). This confirmed that Ga_2_O_3_ in as‐synthesized samples was mostly reduced to Ga^+^ during the reduction process (Ga_2_O_3_ + 2 H^+^Z^−^ + 2 H_2_ → 2 Ga^+^Z^−^ + 3 H_2_O, H_2_O/Ga = 1.5).^[^ ^77^ ^]^ Then Ga^+^ was oxidized to Ga^3+^ species by oxygen pulses (O/Ga = 1).

Three important features were observed in the pulse titration results (Figure 8c): i) On Ga/H‐ZSM‐5 (15, 0.042), no appreciable amount of water or oxygen was formed or consumed, respectively, indicating that Ga species in this sample could not be reduced by H_2_. The oxidation of Ga in this sample remains +3 after the H_2_ treatment at 550 °C, as in the as‐synthesized sample. ii) As the Ga/BAS ratio increased to 0.7, O consumption rose almost linearly with a Ga/O ratio of unity, that is, the majority of Ga^3+^ was reduced to Ga^+^ after H_2_ treatment within this Ga/BAS range. iii) Further increase in the Ga loading did not lead to an increase in the O_2_ consumption, suggesting the presence of a different, redox‐inactive, GaO*_x_* species. This is consistent with the observation that no additional BAS was exchanged in this region, and with the high reduction temperature of Ga_2_O_3_.^[^ ^116^ ^]^

Pulse titration results on Ga supported on Co‐exchanged H‐ZSM‐5 (Ga/Co‐ZSM‐5 (15, Y)), in which all framework Al pairs were occupied by Co^2+^, were similar to those on Ga/H‐ZSM (15, Y) except for a key feature (Figure 8d,e): there was no redox inactive Ga species at very low Ga loading in Ga/Co‐ZSM‐5 (15, 0.05), in contrast to Ga/H‐ZSM‐5 (15, 0.042). This indicates that the redox inactive Ga species in Ga/H‐ZSM‐5 (15, 0.042) occupy framework Al pair sites, and is a divalent cation with Ga in the oxidation state of +3. Considering the 1:1 Ga/BAS exchange ratio in reduced Ga/H‐ZSM‐5 (15, Y), the only possible composition of the redox‐inactive Ga species is Ga_2_O_2_^2+^.^[^ ^54^ ^]^ Based on the titration results, ≈10% of BAS in Ga/H‐ZSM‐5 (15, Y) were exchanged with Ga_2_O_2_^2+^ (Figure 8c), which was lower than the density of Al pairs determined by the Co^2+^ exchange method (≈35%). This discrepancy was rationalized by the fact that there is no clear‐cut definition for framework Al pairs, that is, the cutoff distance between two framework Al atoms beyond which they were not considered “a pair.” This is similar to the NNN and NNNN sites proposed by Phadke et al.^[^ ^100^ ^]^ It is likely that the cutoff distance is different for different divalent cations, leading to different exchange capacities of Co^2+^ and Ga_2_O_2_^2+^.

Ga_2_O_2_^2+^ as a catalytic PDH site in Ga/H‐ZSM‐5 was first proposed by Hensen and co‐workers in a different context. Enhanced PDH activity on Ga/H‐ZSM‐5 was observed when steam was co‐fed with propane, and the Ga_2_O_2_^2+^ moiety was proposed based on the fitting of the extended X‐ray absorption fine structure (EXAFS) spectra.^[^ ^129^ ^]^ As discussed above, the validity of structures of Ga species based on XAS analysis was recently called into question.^[^ ^119^ ^]^ DFT calculations indicated that the key difference between Ga^+^ and Ga_2_O_2_^2+^ was the stability of their oxidized form, that is, [GaO]^+^ and Ga_2_O_2_^2+^, in the reducing environment at high temperatures. The [GaO]^+^ sites can be easily reduced to [Ga]^+^ at high temperatures; while Ga_2_O_2_^2+^ species could dissociate H_2_ to form two isolated [HGa^3+^OH]^+^ interacting via a hydrogen bond, which exhibited lower activation energy for H_2_ recombination compared to isolated [HGa^3+^OH]^+^ (147 vs 242 kJ mol^−1^).^[^ ^130^, ^131^ ^]^ This is consistent with the pulse titration results (Figure 8) that Ga^+^ is redox‐active while Ga_2_O_2_^2+^ cannot be reduced by H_2_ at 550 °C.^[^ ^54^ ^]^ Ga_2_O_2_^2+^ was calculated to be the most stable Ga species around framework Al pairs in MOR zeolites;^[^ ^130^ ^]^ a similar calculation in MFI has yet to be reported.

**Figure 9:**
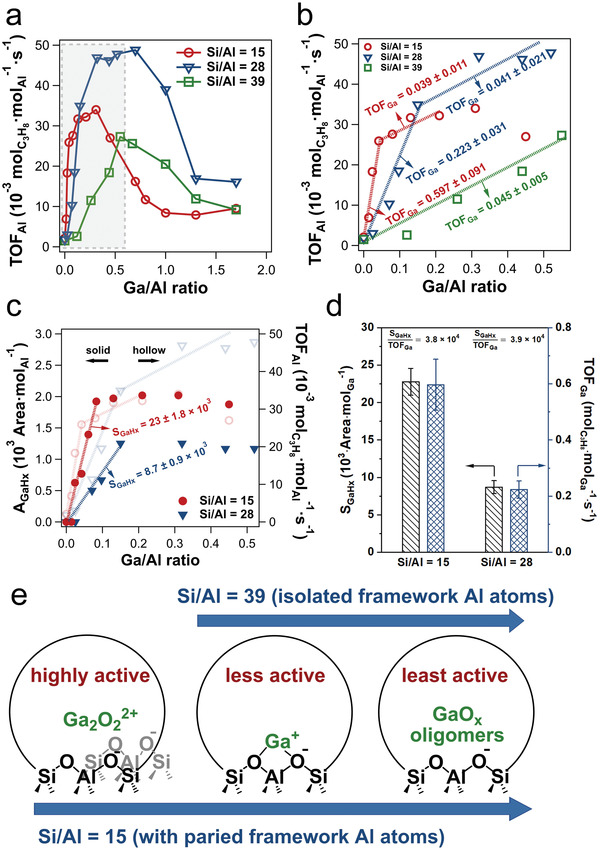
a) TOF_Al_ as a function of the Ga/Al ratio on H‐ZSM‐5 with different Si/Al ratios (15, 28, and 39) and b) zoomed‐in view of low Ga/Al ratios (boxed region in (a)). Reaction conditions: 550 °C; C_3_H_8_ partial pressure, 5.07 kPa with balancing N_2_. The propane conversions are below 5.5% in the rate measurements. c) *A*_GaH_ *_x_* (peak area of GaH*_x_* normalized by a per Al basis determined from infrared spectra) as a function of the Ga/Al ratio on H‐ZSM‐5 with two Si/Al ratios (15 and 28). d) S_GaH_ *_x_* (peak area of GaH*_x_* normalized by a per Ga basis) and TOF_Ga_ (PDH rates normalized by a per Ga basis) between Si/Al ratios of 15 and 28 at a low range of Ga/Al ratios. e) Exchanged Ga species and their PDH performance on reduced Ga/H‐ZSM‐5 with Si/Al ratios of 15 and 39 with the incremental Ga/Al ratios. Arrows indicate increasing Ga loading. a‐e) Adapted with permission.^[^ ^55^ ^]^ Copyright 2021, American Chemical Society.

Ga_2_O_2_^2+^ is the most active PDH species on Ga/H‐ZSM‐5.^[^ ^54^, ^55^, ^110^ ^]^ Based on the pulse titration results, there are three types of Ga species present on reduced catalysts: Ga_2_O_2_^2+^, Ga^+^, and unreduced Ga(III)*_x_* O*_y_* oligomers. On Ga/H‐ZSM‐5 (15, Y), the TOF normalized to the amount of Al in the zeolite (TOF_Al_), increased rapidly at low Ga loadings up to the Ga/Al ratio of 0.05 and then grows more slowly as the Ga/Al ratio rises to 0.3, before declining as the Ga/Al ratio increased further (**Figure** **9**a). A similar 3‐segment dependence of the PDH rate on the Ga/Al ratio was observed on samples with a Si/Al ratio of 28 (Figure 9a). TOF_Al_ on Ga/H‐ZSM‐5 (39, Y) increased almost linearly up to a Ga/Al ratio of 0.55, before declining at higher Ga/Al ratios (Figure 9a). These trends are in general agreement with the results reported by Bell and co‐workers (Figure 6h, Si/Al = 16.5)^[^ ^76^ ^]^ and Lercher and co‐workers (Figure 5a, Si/Al = 50).^[^ ^77^ ^]^ It should be emphasized that the PDH rate is dependent on both Si/Al and Ga/Al ratios.

The intrinsic reaction rates of Ga sites were estimated by determining the slope between TOF_Al_ versus Ga/Al ratio at different Ga/Al ratio ranges, which represented the TOF normalized per unit of added Ga (TOF_Ga_). On samples with a Si/Al ratio of 15, the slope of the TOF_Al_ versus Ga/Al ratio at Ga/Al ratios below 0.042 was 0.60 ± 0.09 mol_C3H8_ mol_Ga_^−1^ s^−1^ (Figure 9b). TOF_Ga_ decreased to 0.04 ± 0.01 in the Ga/Al ratio range of 0.042 to 0.21, smaller than that in the lower Ga/Al range by a factor of ≈15, confirming two Ga sites with distinct PDH activities. Similarly, for samples with a Si/Al ratio of 28, TOF_Ga_ was 0.22 ± 0.03 and 0.04 ± 0.02 mol_C3H8_ mol_Ga_^−1^ s^−1^ in the Ga/Al ratio range of 0–0.15 and 0.15–0.46, respectively. On samples with a Si/Al ratio of 39, TOF_Ga_ was already low (0.05 ± 0.01 mol_C3H8_ mol_Ga_^−1^ s^−1^) at the lowest Ga loading evaluated (Figure 9b). It is noteworthy that the TOF_Ga_ was similar on Ga/H‐ZSM‐5 (15, 0.042‐0.21), Ga/H‐ZSM‐5 (28, 0.15‐0.46), and Ga/H‐ZSM‐5 (39, 0‐0.55); this implies that the same type of Ga species was introduced in all three samples in their respective Ga/Al ratio ranges. Pulse titration results showed that Ga^+^ was introduced in Ga/H‐ZSM‐5 (15, 0.042‐0.21), implying that Ga^+^ was responsible for the measured TOF_Ga_ of ≈0.04 mol_C3H8_ mol_Ga_^−1^ s^−1^. In the low Ga loading range, the integrated area of the GaH*_x_* band grew with the PDH rate in both Ga/H‐ZSM‐5 (15, Y) and Ga/H‐ZSM‐5 (28, Y) (Figure 9c). The ratio between the integrated area of the GaH*_x_* band and TOF_Ga_ in the low Ga loading range was identical within experimental errors in both samples (Figure 11c,d). Spectroscopic and titration results discussed above indicate that GaH*_x_* could only form on Ga_2_O_2_^2+^ (Figures 8 and 9). The heterolytic activation of H_2_ on Ga_2_O_2_^2+^ likely leads to [H‐GaOGa(OH)]^2+^, which is consistent with the absence of [GaD_2_]^+^ species (Figure 4c,d).

In addition to Ga/H‐ZSM‐5, Ga exchanged on CHA^[^ ^56^ ^]^ has also been studied. Lobo and co‐workers^[^ ^56^ ^]^ investigated the Ga speciation and PDH of Ga/H‐CHA and found that only extra‐framework Ga^+^ sites were formed upon reduction of Ga/H‐CHA catalyst, independent of Si/Al ratios. Isolated Ga^+^ sites reacted reversibly with H_2_ to form GaH*_x_* (2034 cm^−1^) at 150 °C and decomposed at high temperatures (550 °C). Thus, it remained unclear whether the GaH*_x_* band on Ga/H‐CHA corresponded to Ga_2_O_2_^2+^ as in Ga/H‐ZSM‐5. The absence of Ga_2_O_2_^2+^ in Ga/H‐CHA at a high temperature suggested that the framework could not stabilize Ga_2_O_2_^2+^ at reaction conditions, which agreed with the much lower TOF_Ga_ on Ga/H‐CHA (Table 1).^[^ ^56^ ^]^ Correlations between GaH*_x_* versus Ga/Al ratio, as well as PDH rate versus Ga/Al ratio, implied that extra‐framework Ga^+^ was more likely the active center catalyzing PDH.

Lercher and co‐workers^[^ ^132^ ^]^ recently reported a highly selective Ga‐modified BEA zeolite catalyst for PDH with 82% selectivity for propylene at 19% propane conversion. The active sites have been identified as dehydrated and tetrahedrally coordinated Ga^3+^ in the *BEA framework. Detailed kinetic analysis indicated the different RDS at different propane partial pressures: PDH rate was determined by the first C—H bond cleavage at low propane partial pressures, while the rate was limited by the H_2_ desorption at high propane partial pressures.

### 2.2 Gallium's Role in PDH Mechanism

To date, proposed PDH mechanisms on Ga/H‐ZSM‐5 can be broadly categorized into carbenium and alkyl mechanisms.^[^ ^76^, ^77^, ^133^, ^134^ ^]^ The carbenium mechanism claims that C_3_H_8_ is initially activated on the BAS to form a carbenium ion, and the role of Ga is to facilitate the recombination of the H atom on the catalyst surface to form H_2_, thereby increasing the reaction rate.^[^ ^133^, ^134^ ^]^ Meanwhile, Ga species activate C_3_H_8_ directly with the formation of Ga‐C_3_H_7_ as the intermediate in the alkyl mechanism, followed by the *β*‐H elimination of Ga‐C_3_H_7_ to form C_3_H_6_ and Ga‐H species, and closed by the desorption of H_2_ via proton transfer.^[^ ^76^, ^77^ ^]^ In this section, we discuss the experimental and computational evidence supporting the two mechanisms.

#### 2.2.1 Carbenium Mechanism

**Figure 10:**
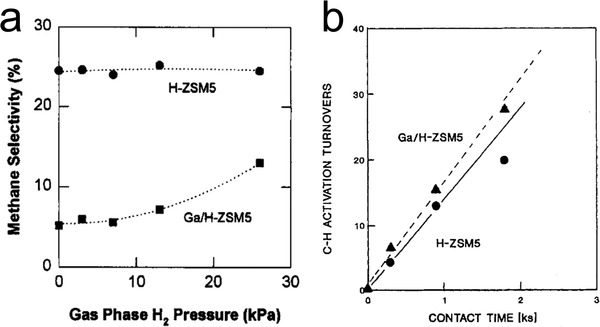
a) Methane selectivity of propane dehydrocyclodimerization as a function of H_2_ pressure on H‐ZSM‐5 and Ga/H‐ZSM‐5 catalyst. Adapted with permission.^[^ ^133^ ^]^ Copyright 1996, Elsevier. b) C—H bond activation turnovers versus contact time. Reaction conditions in (a): 500 °C, 26.6 kPa C_3_H_8_. Reaction conditions in (b): 500 °C, 3.56 kPa C_3_H_8_, and 3.10 kPa C_3_D_8_. Adapted with permission.^[^ ^135^ ^]^ Copyright 1993, Springer Nature.^[^ ^133^, ^135^ ^]^

Iglesia and co‐workers reported that the presence of Ga led to the increase of the propane turnover rates and the decrease of the cracking selectivity compared to H‐ZSM‐5,^[^ ^99^, ^133^, ^134^, ^135^, ^136^ ^]^ and attributed this enhancement to Ga's role of re‐combinative desorption of H atoms formed in the dehydrogenation steps. The dependence of cracking selectivity (or methane selectivity) on H_2_ partial pressure determined on Ga/H‐ZSM‐5 and H‐ZSM‐5 showed contrasting trends (**Figure** **10**a). H‐ZSM‐5 showed a high selectivity for cracking, attributed to the catalyst's limited capacity for removing hydrogen adatoms produced in the dehydrogenation to form gas phase H_2_. The accumulating hydrogen adatoms led to high surface hydrogen fugacity, and thus drove the formation of methane, the most H‐rich hydrocarbon, via cracking.^[^ ^133^ ^]^ Recombinative desorption of H adatoms and dissociative adsorption of H_2_ are the same reaction in opposite directions. The inability of H‐ZSM‐5 to facilitate this equilibrium decouples the surface reactions from the H_2_ partial pressure, leading to the lack of H_2_ partial pressure dependence of methane selectivity. In contrast, the cracking selectivity of Ga/H‐ZSM‐5 increased along with the H_2_ partial pressure (Figure 10a). Ga species were proposed to function as a “porthole” between the adsorbed H adatoms and the gas phase H_2_, that is, establishing the equilibrium between adsorbed H and gas phase H_2_. Thus, higher H_2_ partial pressure led to higher surface H fugacity, which favors the cracking pathway.

H‐D scrambling experiments provided additional insights into Ga's role in the PDH mechanism.^[^ ^133^, ^135^, ^136^ ^]^ Mixtures of C_3_H_8_ and C_3_D_8_ were employed to measure the C—H activation rate by determining the rate of H‐D scrambling. Similar rates were determined on both H‐ZSM‐5 and Ga/H‐ZSM‐5 (Figure 10b), suggesting a similar ability for C—H activation on both catalysts. Importantly, measured rates for C—H activation were significantly higher than PDH by a factor of ≈20 for H‐ZSM‐5 and of ≈10 for Ga/H‐ZSM‐5,^[^ ^135^ ^]^ which were interpreted as evidence for the PDH mechanism involving a pseudo‐equilibrated C—H activation step followed by the rate‐determining H_2_ desorption. Similar observations were reported on Zn/H‐ZSM‐5 and Co/H‐ZSM‐5 catalysts, suggesting the generality of this mechanism on metal‐exchanged catalysts.^[^ ^95^, ^135^, ^136^, ^137^ ^]^ However, C—H activation in H‐D scrambling may not proceed via the same mechanism or involve the same reaction intermediates as in PDH, for example, the H‐D exchange of methane was proposed to occur on acid sites in zeolites without the need for complete scission of the C—H bond.^[^ ^138^, ^139^ ^]^ Moreover, H‐D scrambling occurred on a Pt‐based single‐atom catalyst below 200 °C, which was more than 300 °C lower than the PDH temperature.^[^ ^42^ ^]^

**Scheme 1:**
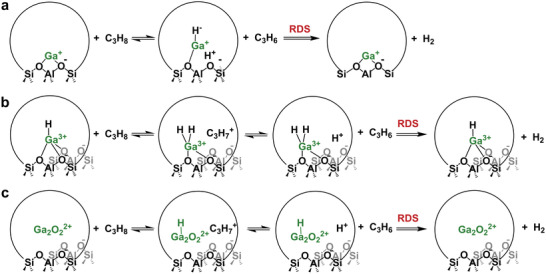
Schematic of proposed carbenium mechanisms for the PDH on a) Ga^+^, b) [GaH]^+^, and c) Ga_2_O_2_^2+^.

The central tenet of the carbenium mechanism involves the rapid formation of a [C_3_H_7_]^+^ intermediate and slow H_2_ desorption.^[^ ^133^, ^140^ ^]^ For both H‐ZSM‐5 and Ga/H‐ZSM‐5, propane is initially activated by H^+^ to form the carbenium ion, followed by *β*‐H elimination to release propylene and hydrogen. The latter step is accelerated by the presence of Ga species. Although the active Ga species in the zeolite remains debated,^[^ ^54^, ^55^, ^76^, ^102^, ^133^ ^]^ including isolated Ga^+^ and [GaH]^2+^ and [Ga_2_O_2_]^2+^, similar mechanisms to those proposed by Iglesia and co‐workers could be envisioned on these proposed active structures, as illustrated in **Scheme** **1**.

#### 2.2.2 Alkyl Mechanism

**Figure 11:**
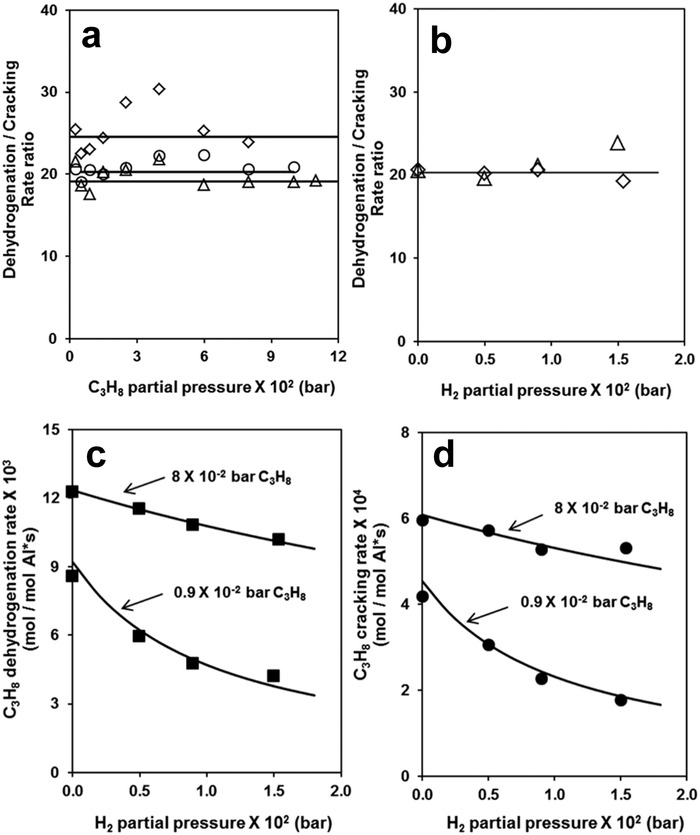
The ratio of dehydrogenation to cracking rates (D/C) of Ga/H‐ZSM‐5 as a function of a) C_3_H_8_ partial pressure at 718, 733, and 753 K, b) H_2_ partial pressure at 733 K. In (a), triangles, circles, and diamonds indicate the ratios of rates at 718, 733, and 753 K, respectively. In (b), open triangles and open diamonds indicate the D/C ratios measured at 0.9 × 10^−2^ bar and 8 × 10^−2^ bar C_3_H_8_, respectively. c) C_3_H_8_ dehydrogenation rates and d) cracking rates at 733 K as a function of H_2_ partial pressure. All rates were extrapolated to zero space‐time. a‐d) Adapted with permission.^[^ ^76^ ^]^ Copyright 2019, American Chemical Society.

Different from the carbocation intermediate and H_2_ desorption RDS in the carbenium mechanism, Bell and co‐workers proposed that the activation of the C—H bond in propane leading to the formation of [C_3_H_7_‐GaH]^+^ was the RDS in PDH.^[^ ^76^ ^]^ The ratio of dehydrogenation to cracking (D/C) was independent of C_3_H_8_ or H_2_ partial pressure (**Figure** **11**a,b), which was interpreted as evidence for both dehydrogenation and cracking reactions occurring on the same active sites in Ga/H‐ZSM‐5 and involving the same surface intermediate, that is, [C_3_H_7_‐GaH]^+^. Negative reaction orders of H_2_ were determined resulting in both dehydrogenation and cracking reactions (Figure 11c,d), consistent with previous results,^[^ ^133^ ^]^ with the inhibition more severe at lower propane partial pressures. It was proposed that H_2_ competed with C_3_H_8_ for adsorption on the active sites ([GaH]^2+^), and the inhibition became weaker at higher propane partial pressure due to the strong interaction between the alkyl and active Ga species. The mechanism proposed by Lercher and co‐workers, based primarily on computational results, is consistent with key aspects of the alkyl mechanism, though the assumed structure of active sites is different.^[^ ^77^ ^]^

**Figure 12:**
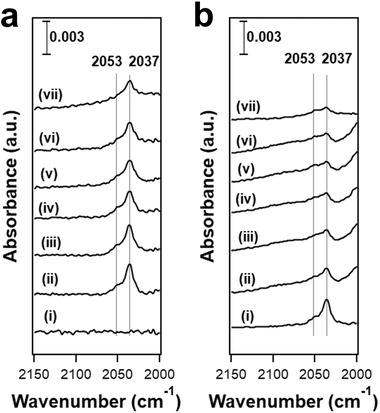
a) FTIR spectra of Ga/H‐ZSM‐5 (15, 0.13): i) before H_2_ treatment and ii–vii) upon evacuating H_2_ for 2, 4, 6, 8, 10, and 30 min. b) C_3_H_8_ treatment on reduced Ga/H‐ZSM‐5 (15,0.13): i) before C_3_H_8_ treatment; ii–vi) C_3_H_8_ treatment (0.27 kPa) for 2, 4, 6, 8, and 10 min; vii) after evacuation. a,b) Adapted with permission.^[^ ^55^ ^]^ Copyright 2021, American Chemical Society.

The competitive adsorption of C_3_H_8_ and H_2_ on active Ga species was supported by Xu and co‐workers' investigations using in situ FTIR spectroscopy.^[^ ^55^ ^]^ Upon hydrogen reduction of Ga/H‐ZSM‐5 at 550 °C, GaH*_x_* bands appeared at 2037 cm^−1^ with a shoulder at 2053 cm^−1^ (**Figure** **12**aii). The intensity of GaH*_x_* bands decreased faster in the presence of propane than under evacuation (Figure 12a,b), indicating that propane accelerated the decomposition of GaH*_x_*. This implies that at least a fraction of hydrogen and propane competes to adsorb on the same sites. The apparent activation energy of GaH*_x_* decomposition in the presence of propylene is comparable to the minimal apparent activation of PDH, suggesting that the desorption of adsorbed H could also be a kinetically relevant step in PDH (in addition to the initial C—H bond activation step).

**Scheme 2:**
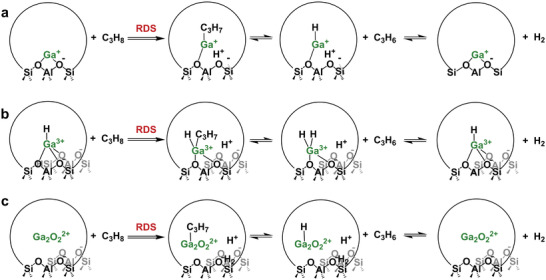
Schematic of proposed alkyl mechanisms for the PDH on a) Ga^+^, b) [GaH]^+^, and c) Ga_2_O_2_^2+^.

Similar to the carbenium mechanism, the alkyl mechanism could be at play regardless of the specific structure of active Ga species, on which propane is initially activated by the metal cation to form the alkyl gallium intermediate, followed by the *β*‐elimination and the desorption of propylene and H_2_. The C—H activation step and the formation of an alkyl metal intermediate were proposed as the RDS, as shown in **Scheme** **2**.

#### 2.2.3 Carbenium Mechanism versus Alkyl Mechanism

The key difference between the two mechanisms is the role of Ga species in PDH: facilitate the recombination of H adatoms to release H_2_ versus activate the C—H bond in propane. Both mechanisms are consistent with the cracking/dehydrogenation ratio in their respective ranges of H_2_ pressure (Figures 10a and 11b).^[^ ^76^, ^133^ ^]^ The H_2_ pressure range covered by Iglesia and co‐workers' report (0–25 kPa) was broader than that of Bell and co‐workers' (0–1.5 kPa) by roughly a factor of ten, suggesting that the reaction mechanism proposed by the former was applicable in a wider parameter space. Bell and co‐workers reported that the PDH rate was roughly 1st order at low propane partial pressure and 0th order at higher pressure,^[^ ^76^ ^]^ the latter which corresponded to the region of active sites saturated by adsorbed propane. The carbenium mechanism also predicts a similar trend of propane reaction order, though the transition from positive to zero reaction order may occur at a different propane partial pressure. Thus, kinetic results alone are unlikely to unambiguously differentiate the two mechanisms.

**Figure 13:**
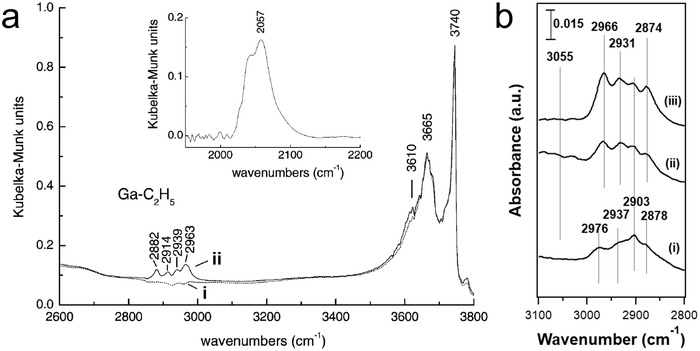
a) DRIFTS spectra of Ga/ZSM‐5: i) Ga/ZSM‐5 reduced in hydrogen and evacuated at 500 °C; and ii) after heating in 0.93 kPa ethane to 250 °C followed by evacuation and cooling to room temperature. Adapted with permission.^[^ ^141^ ^]^ Copyright 2005, Royal Society of Chemistry. b) FTIR spectra of C_3_H_8_ treatment on Ga/H‐ZSM‐5 with Ga/Al ratios of i) 0, ii) 0.042, and iii) 0.13. Adapted with permission.^[^ ^55^ ^]^ Copyright 2021, American Chemical Society.

Another potentially experimentally verifiable difference between mechanisms is the formation of an alkyl metal intermediate, Ga‐C_3_H_7_, whose C—H stretching bands in IR spectroscopy are expected to be different from those in C_3_H_7_^+^. Hensen and co‐workers applied DRIFTS to investigate the ethane dehydrogenation pathway on Ga/H‐ZSM‐5 and observed the C—H stretching modes attributable to Ga‐C_2_H_5_ (**Figure** **13**a).^[^ ^141^ ^]^ This was confirmed by Yuan et al.'s results of propane in both H‐ZSM‐5 and Ga/H‐ZSM‐5 (Figure 13b).^[^ ^55^ ^]^ On H‐ZSM‐5, propane treatment led to the appearance of four peaks centered at 2976, 2937, 2903, and 2878 cm^−1^ (Figure 13bi): assigned to antisymmetric CH_3_, antisymmetric CH_2_, symmetric CH_3_, and symmetric CH_2_ stretching modes, respectively.^[^ ^142^, ^143^, ^144^ ^]^ Three peaks distinct from those on H‐ZSM‐5 centered at 2966, 2931, and 2874 cm^−1^ were detected on Ga/H‐ZSM‐5 with two different Ga loadings (Figure 13bii,iii), that is, the presence of Ga alters the C—H stretching bands. Interpreting these subtle differences demands caution, as the local environment of adsorbed hydrocarbons could affect spectral features. The possibility that both Ga‐C_3_H_7_ and C_3_H_7_^+^ are present and in turn, both the carbenium and the alkyl mechanisms are at play, cannot be ruled out. Thus, PDH could occur via different mechanisms on different types of Ga sites. Since the distribution of different Ga species is impacted by both Ga loading and Al distribution in H‐ZSM‐5, it is possible that the carbenium or alkyl mechanism is dominant on Ga/H‐ZSM‐5 with different densities of Ga species.

DFT calculations have also been extensively employed to compare the carbenium and alkyl mechanisms,^[^ ^77^, ^102^, ^145^, ^146^, ^147^, ^148^ ^]^ but without a clear consensus. Pidko et al. examined the ethane activation pathway using Ga^+^, [GaH_2_]^+^, and [GaH]^2+^ as models in reduced Ga/H‐ZSM‐5 catalysts. By comparing the energies of the initial C—H activation in ethane at these sites, it was concluded that the reaction most likely occurred at the Ga^+^ site via the alkyl route. Meanwhile, both [GaH_2_]^+^ and [GaH]^2+^ sites showed high activation energies.^[^ ^145^ ^]^ Mansoor et al. also examined the mechanism of ethane dehydrogenation reaction on Ga^+^, [GaH_2_]^+^, and [GaH]^2+^ using larger cluster models with different Al pair distances.^[^ ^102^ ^]^ They found [GaH_2_]^+^ and [GaH]^2+^ were more active in ethane dehydrogenation than Ga^+^. Interestingly, the RDS was found to depend on the reaction pathway and proposed active sites. For [GaH_2_]^+^, the stepwise alkyl pathway is more favorable, with C‐H activation being the RDS. For the [GaH]^2+^, ethene formation is the RDS when the reaction proceeds via the alkyl pathway, however, the carbenium pathway is slightly more favorable with a similar ethene formation and C—H activation energy. Schreiber et al. compared propane activation over BAS (H^+^), Ga^+^, and Ga^+^–H^+^ pair, and found both H^+^ and Ga^+^ show a higher activation energy of propane than the Ga^+^–H^+^ pair.^[^ ^77^ ^]^ The Ga^+^–H^+^ pair is likely to catalyze PDH via the alkyl pathway. Computational investigations of propane activation mechanism on the newly identified Ga_2_O_2_^2+^ supported on MFI have yet to be reported and could be an interesting direction to explore in future studies.

## 3 Concluding Remarks and Perspectives

The structural complexity of zeolite‐supported metal catalysts affords them rich chemistry and unique catalytic properties. Despite best synthetic efforts, metals are typically present in the zeolite in multiple forms, for example, metal cations, cationic metal hydroxide or oxide species, and neutral hydroxide and oxide species. Elucidating metal speciation in zeolite is challenging but a prerequisite for identifying active structures and understanding reaction mechanisms. We took Ga/H‐ZSM‐5 as an example to highlight that technological advances and conceptual innovations could deepen the understanding of structure–activity relations. The employment of advanced in situ characterization techniques, for example, XANES, provided foundational knowledge regarding how Ga/H‐ZSM‐5 was activated in the reducing environment, while quantitative techniques, such as FTIR spectroscopy and pulse titration, enabled the determination of the density of multiple Ga species. The recognition of framework Al distribution as a relevant variable in PDH activity introduced a new dimension in understanding the Ga speciation, which led to the discovery of Ga_2_O_2_^2+^ as the most active species in the PDH.

In light of the crucial role of framework Al pairs in H‐ZSM‐5 to stabilize Ga_2_O_2_^2+^, the ability to synthesize Al‐pair‐rich H‐ZSM‐5 could increase the density of Ga_2_O_2_^2+^. Gounder and co‐workers^[^ ^127^, ^128^, ^149^ ^]^ employed organic and inorganic structure‐directing agents to tune the Al distribution in CHA and MFI zeolites, which offers a viable path to optimize the PDH activity of Ga/H‐ZSM‐5. Reducing the diffusion length of propane and propylene from the active Ga species could also enhance PDH activity. Wannapakdee et al.^[^ ^150^ ^]^ prepared Ga/H‐ZSM‐5 nanosheets, and found that the propane conversion is three times higher than that of conventional zeolites. Their follow‐up work indicated that this strategy was effective for *n*‐pentane conversion.^[^ ^151^ ^]^

Ga/H‐ZSM‐5 catalysts show excellent stability in PDH at low conversions (<10%).^[^ ^55^, ^76^ ^]^ The stability of Ga/H‐ZSM‐5 for PDH has been less studied at higher conversions. This is primarily because the rates were measured at low conversions to obtain the intrinsic reaction rates required to establish structure–activity relationships. At higher propane conversions, side reactions, such as cracking, oligomerization, aromatization, and carbon deposition, can also occur due to BAS, which are common in hydrocarbon catalysis on acidic zeolite catalysts at high temperatures.^[^ ^97^, ^120^, ^152^ ^]^ Fabricating zeolites with better diffusion properties, for example, with hierarchical structures and in the nanosheet form, has been shown to be an effective strategy for enhancing the catalyst stability,^[^ ^97^ ^]^ which could be leveraged to enhance the stability of Ga/H‐ZSM‐5 in PDH.^[^ ^153^, ^154^ ^]^

Ga_2_O_2_^2+^ was predicted by DFT calculations more than a decade before its experimental discovery, highlighting the potential of a combined experimental and computational approach in determining the structure of the active center. A few strategies employed in the mechanistic studies of Ga/H‐ZSM‐5 catalyzed PDH could help determine active structures in metal‐exchanged zeolite catalysts in general: 1) Determining the BAS consumption versus metal/Al ratios. The relationship between BAS consumption versus metal/Al ratios is essential in estimating the density of cationic metal species entered into metal‐exchanged zeolite catalysts via ion‐exchange. Although multiple methods are available to determine the BAS densities, infrared spectroscopy with pyridine as the probe molecule is the most reliable and can be conducted on catalysts after in situ pretreatment. 2) Quantitative pulse reactions could determine the oxidation state of metal species and differentiate different species based on their redox properties. 3) Employing probe molecules, for example, H_2_, H_2_O, and CO, in spectroscopic studies could be informative in distinguishing different active structures. 4) Correlating trends in intrinsic rates and *E*_app_ with densities of multiple active sites could help determine the most active structure.

## Conflict of Interest

The authors declare no conflict of interest.
